# Tracking the Electron Transfer Cascade in European Robin Cryptochrome 4 Mutants

**DOI:** 10.1021/jacs.3c00442

**Published:** 2023-05-17

**Authors:** Daniel Timmer, Anders Frederiksen, Daniel C. Lünemann, Anitta R. Thomas, Jingjing Xu, Rabea Bartölke, Jessica Schmidt, Tomáš Kubař, Antonietta De Sio, Ilia A. Solov’yov, Henrik Mouritsen, Christoph Lienau

**Affiliations:** † Institut für Physik, Carl von Ossietzky Universität, 26129 Oldenburg, Germany; ‡ Institut für Biologie und Umweltwissenschaften, Carl von Ossietzky Universität, 26129 Oldenburg, Germany; § Department for Theoretical Chemical Biology, Institute for Physical Chemistry, Karlsruhe Institute of Technology, Kaiserstrasse 12, 76131 Karlsruhe, Germany; ∥ Research Centre for Neurosensory Science, Carl von Ossietzky Universität, 26111 Oldenburg, Germany; ⊥ Center for Nanoscale Dynamics (CENAD), Carl von Ossietzky Universität Oldenburg, Institut für Physik, 26129 Oldenburg, Germany

## Abstract

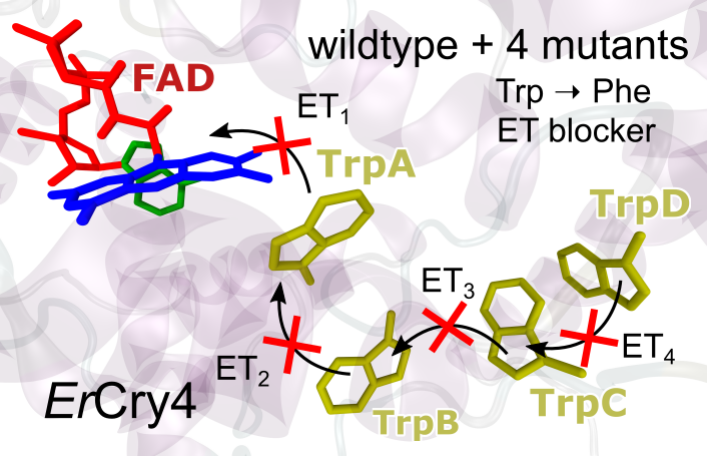

The primary step in the mechanism by which migratory birds sense the Earth’s magnetic field is thought to be the light-induced formation of long-lived magnetically sensitive radical pairs within cryptochrome flavoproteins located in the birds’ retinas. Blue-light absorption by the non-covalently bound flavin chromophore triggers sequential electron transfers along a chain of four tryptophan residues toward the photoexcited flavin. The recently demonstrated ability to express cryptochrome 4a from the night-migratory European robin (*Erithacus rubecula*), *Er*Cry4a, and to replace each of the tryptophan residues by a redox-inactive phenylalanine offers the prospect of exploring the roles of the four tryptophans. Here, we use ultrafast transient absorption spectroscopy to compare wild type *Er*Cry4a and four mutants having a phenylalanine at different positions in the chain. We find that each of the three tryptophan residues closest to the flavin adds a distinct relaxation component (time constants: 0.5, 30, and 150 ps) in the transient absorption data. The dynamics of the mutant containing a phenylalanine at the fourth position, furthest from the flavin, are very similar to those of wild type *Er*Cry4a, except for a reduced concentration of long-lived radical pairs. The experimental results are evaluated and discussed in the framework of real-time quantum mechanical/molecular mechanical electron transfer simulations based on the density functional-based tight binding approach. This comparison between simulation results and experimental measurements provides a detailed microscopic insight into the sequential electron transfers along the tryptophan chain. Our results offer a route to the study of spin transport and dynamical spin correlations in flavoprotein radical pairs.

## 1 Introduction

Cryptochromes (Cry) are blue-light-sensitive flavoproteins that have a variety of functions.^1−4^ They are essential, for example, for maintaining circadian rhythms in animals^5,6^ and plants^7^ and for controlling aspects of plant growth^8^ and flowering.^9^ Moreover, there is increasing,^10^ albeit so far indirect, evidence that cryptochromes are involved in the mechanism that allows migratory songbirds to sense the direction of the Earth’s magnetic field.^11−14^

Some cryptochromes^10,15,16^ non-covalently bind a flavin adenine dinucleotide (FAD) chromophore linked to the surface of the protein by a ∼2.5 nm chain of aromatic amino acid residues. In plant Crys, the chain comprises three tryptophans (Trp),^12,17^ while in animal and animal-like Crys, there are often four tryptophans.^10,18^ Blue light absorption via a π → π* transition in the isoalloxazine (flavin) moiety of the fully oxidized FAD_ox_ ground state triggers a series of electron transfers along the Trp chain and results in the formation of a pair of radicals, [FAD^•–^TrpH^•+^], in a spin-correlated electronic singlet state.^10,19,20^ The lifetime of this state (denoted ^S^RP1), which is typically in the microsecond range,^10,11,21^ should be long enough to allow a weak magnetic field to modulate the efficiency of coherent interconversion with the corresponding triplet state (^T^RP1).^12,22−24^ This process is mainly induced by hyperfine couplings and thus depends on the alignment of nuclear spins coupled to the unpaired electrons.^12,18,23^ RP1 is anticipated to be sensitive to the direction of an external magnetic field and, therefore, to be a suitable candidate for the magnetic compass sensor in birds.^10^ RP1 deprotonates on a microsecond time scale, thereby forming a longer-lived, magnetically insensitive, secondary radical pair, [FAD^•–^Trp^•^].^21^ Protonation of FAD^•–^, to give FADH^•^, and reduction of Trp^•^ further stabilize the reduced flavin and lead to the formation of a signaling state with an anisotropic quantum yield that encodes the direction of the magnetic field. The signaling state returns to the fully oxidized ground state on a much longer time scale. This light-dependent radical-pair mechanism is thought to be sufficiently sensitive to provide night-migratory birds with magnetic compass information even under dim light conditions.^11,25^

Light-induced electron transfer along the Trp chain is thus the primary step in the photochemical cycle of cryptochromes. The ultrafast dynamics of flavins and flavoproteins have been the subject of numerous experimental studies during the last two decades.^19,26−36^ In FAD in aqueous solution, an intramolecular electron transfer from the adenine to the excited flavin occurs in a stacked conformation with a time constant of 5 ps, followed by charge recombination on a faster time scale.^31^ In the open conformation, at low pH, a much longer, nanosecond lifetime has been measured.^31^ For protein-bound FAD, rapid electron transfer from a nearby tryptophan residue forms a [FAD^•–^TrpH^•+^] radical pair within ∼1 ps in animal type I Crys^19,29,37^ and with sub-picosecond time constants in plant Crys and photolyases.^28,38−41^ Dynamics on longer time scales are mainly interpreted in terms of the sequential electron transfer steps along the Trp chain. It remains difficult, however, to unambiguously assign rate constants to the individual charge separation steps due to competing nonequilibrium processes such as vibrational cooling.^29^

Very recently, it became possible to recombinantly express and purify^42^ wild type cryptochrome 4a from the night-migratory European robin^10^ (*Er*Cry4a) with more than 97% FAD-bound.^10^ Transient absorption spectra of wild type *Er*Cry4a and four mutants, in which one of the tryptophans involved in the electron transfer had been site-selectively replaced by a redox-inactive phenylalanine (Phe), were recorded with sub-nanosecond time resolution.^10^ The study gave evidence for the creation of light-induced radicals with lifetimes exceeding 100 ns in the wild type protein and in the mutant in which the terminal Trp had been replaced by Phe. The experiments also showed changes in the transient absorption signals of up to 15% produced by ∼10 mT magnetic fields. Due to the limited time resolution, the sequential charge transfer steps could not be resolved experimentally but were estimated computationally based on molecular dynamics (MD) simulations and empirical Moser–Dutton theory. Since those rates are critical for understanding the magnetic sensitivity of *Er*Cry4a, their independent measurement using ultrafast spectroscopy is urgently needed. Such experiments would open new frontiers for probing intraprotein charge and spin transfer dynamics^43^ and provide important opportunities for experiment/theory comparison.^10,18,44,45^

In the present study, we provide a comparative ultrafast optical study of the Trp-tetrad electron transfer cascade in wild type *Er*Cry4a and in the four Trp → Phe mutants of *Er*Cry4a. This approach allows us to isolate the dynamics and yields of the first three electron transfers in *Er*Cry4a. It provides a benchmark for modeling radical pair formation in cryptochromes, as explored here by a direct comparison with hybrid quantum-mechanics/molecular mechanics simulations. The emerging synergy between the experiment and simulations provides a first step toward quantitative modeling of electron transport in *Er*Cry4a and forms a basis for the investigation of spin transport and correlations in magnetically sensitive flavin-tryptophan radical pairs.

## 2 Results

### 2.1 Spectroscopic Properties of *Er*Cry4a Mutants

**Figure 1:**
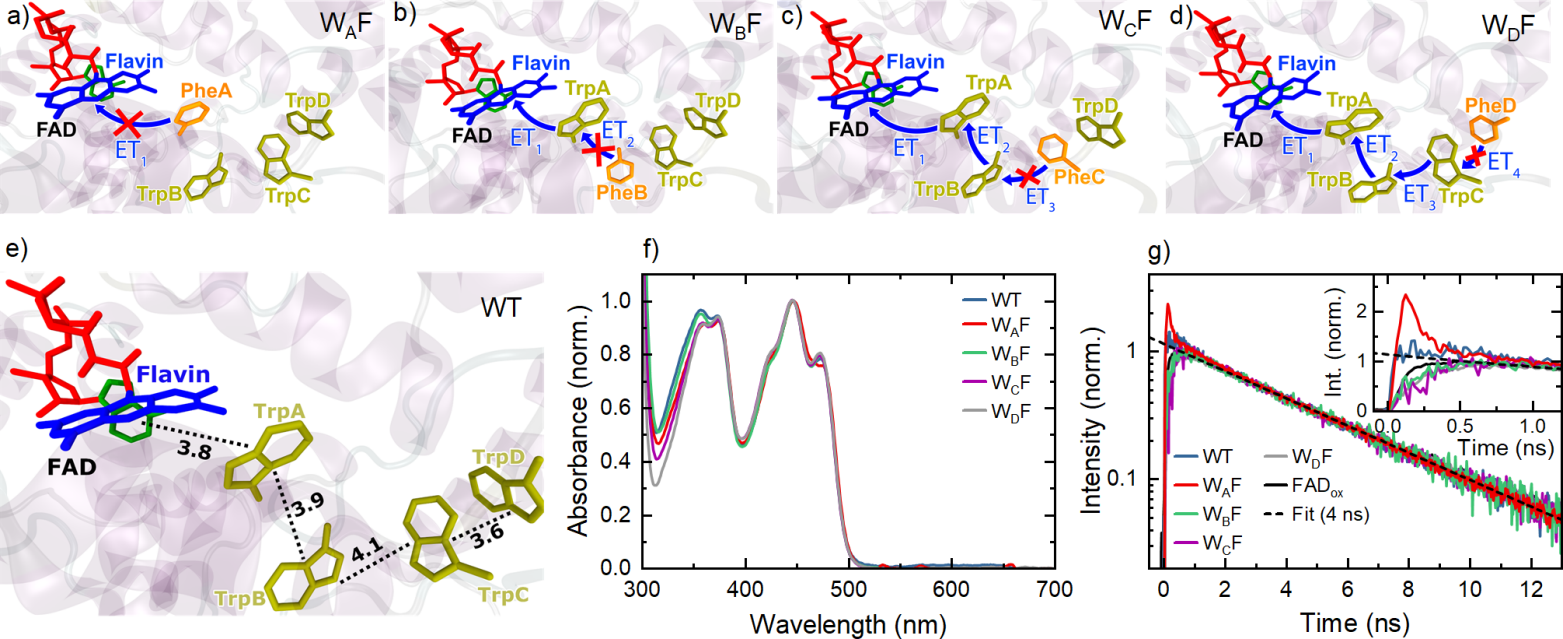
Visualization of the principal electron transfer route in *Er*Cry4a and the effect of single amino acid mutations in blocking the electron transfer process at different stages. (a–d) Electron transfers in *Er*Cry4a mutants involving Trp → Phe substitutions with W395 (Trp_A_), W372 (Trp_B_), W318 (Trp_C_), and W369 (Trp_D_). The arrows illustrate the electron transfer paths, while the crossed arrows represent the blocked electron transfers, as suggested by the experimental data in this study. (e) Edge-to-edge distances between the FAD chromophore and the components of the Trp-tetrad in wild type (WT) *Er*Cry4a. (f) Normalized UV–visible absorption spectra of fully oxidized wild type *Er*Cry4a and its four mutants. The pronounced vibronic structure on the 450 nm band indicates that the FAD is correctly bound to the protein. (g) Transient photoluminescence decay curves of wild type *Er*Cry4a and its mutants compared to that of free FAD_ox_ in buffer solution (solid lines) together with a mono-exponential, 4 ns decay (dashed line). Only the W_A_F mutant shows an initial fast component originating from the weak emission of protein-bound FAD (inset).

Spectroscopic studies were performed on wild type *Er*Cry4a and four Trp → Phe mutants expressed and purified as described earlier.^10^ The structure of *Er*Cry4a has not yet been determined due to the difficulty of obtaining crystals of sufficient quality. Gene sequencing^46^ and computational modeling^10,46^ suggest that it is similar to that of pigeon (*Columba livia*, *Cl*) Cry4, which also has four tryptophans involved in photo-activation, namely, Trp_A_ (W395), Trp_B_ (W372), Trp_C_ (W318), and Trp_D_ (W369).^16^ The structure of the FAD chromophore and the Trp tetrad, as obtained from such simulations, is shown in Figure 1e. Site-specific mutagenesis^10^ has been used to express four mutants, W_X_F (X = A, B, C, D), in which one of the four tryptophans (W) has been selectively replaced by phenylalanine (F) to block the electron transfer at different positions along the chain, as illustrated in Figure 1a–d.

The UV–visible absorption spectra of wild type *Er*Cry4a and its four mutants are very similar (Figure 1f), featuring a main peak centered at 450 nm with a well-resolved vibronic structure. Recent hybrid quantum/classical modeling,^47^ explicitly treating couplings to nuclear vibrations, have described the structure of these spectra and assigned the 450 nm band to a π → π* transition involving the π_2_ and π_3_ orbitals localized on the isoalloxazine moiety of the FAD cofactor.^48^ The vibronic structure is a distinct sign of chromophore binding to the protein and vanishes for free FAD in solution. The structured peak around 370 nm arises from the π_1_ → π_3_ transition.^47^

All five proteins showed weak photoluminescence (PL) with an unstructured emission around 550 nm (see Figure S1). The corresponding excitation spectra are similar and independent of the emission energy (Figure S1). In contrast to the absorption spectra (Figure 1f), the excitation spectra of all five proteins showed an absorption peak with no vibronic structure in the 450 nm band similar to that seen for free FAD in buffer solution (Figure S1). Comparison of the PL emission intensities of the proteins and of free FAD indicates that, in all of the protein samples, >97% of all FAD molecules are bound and that the weak residual PL seen in Figure S1 stems from a small amount of unbound FAD. This observation was confirmed by time-resolved PL studies (Figure 1g) showing, for all proteins except W_A_F, a 4-ns mono-exponential decay matching that of free FAD in buffer solution. The W_A_F mutant revealed an additional fast, resolution-limited emission component (Figure 1g, inset). In this mutant, Trp_A_, the closest tryptophan to the FAD, had been replaced by Phe, preventing the normally rapid electron transfer from Trp_A_ to FAD. Therefore, the optically excited FAD_ox_^*^ in W_A_F is longer-lived and its emission is not completely quenched by electron transfer. In all other mutants, the Trp_A_ electron donor is present, resulting in efficient PL quenching and hence no emission from FAD_ox_^*^. This observation already points to an efficient electron transfer from Trp_A_ to FAD_ox_.

### 2.2 Transient Absorption Spectra of *Er*Cry4a Proteins

**Figure 2:**
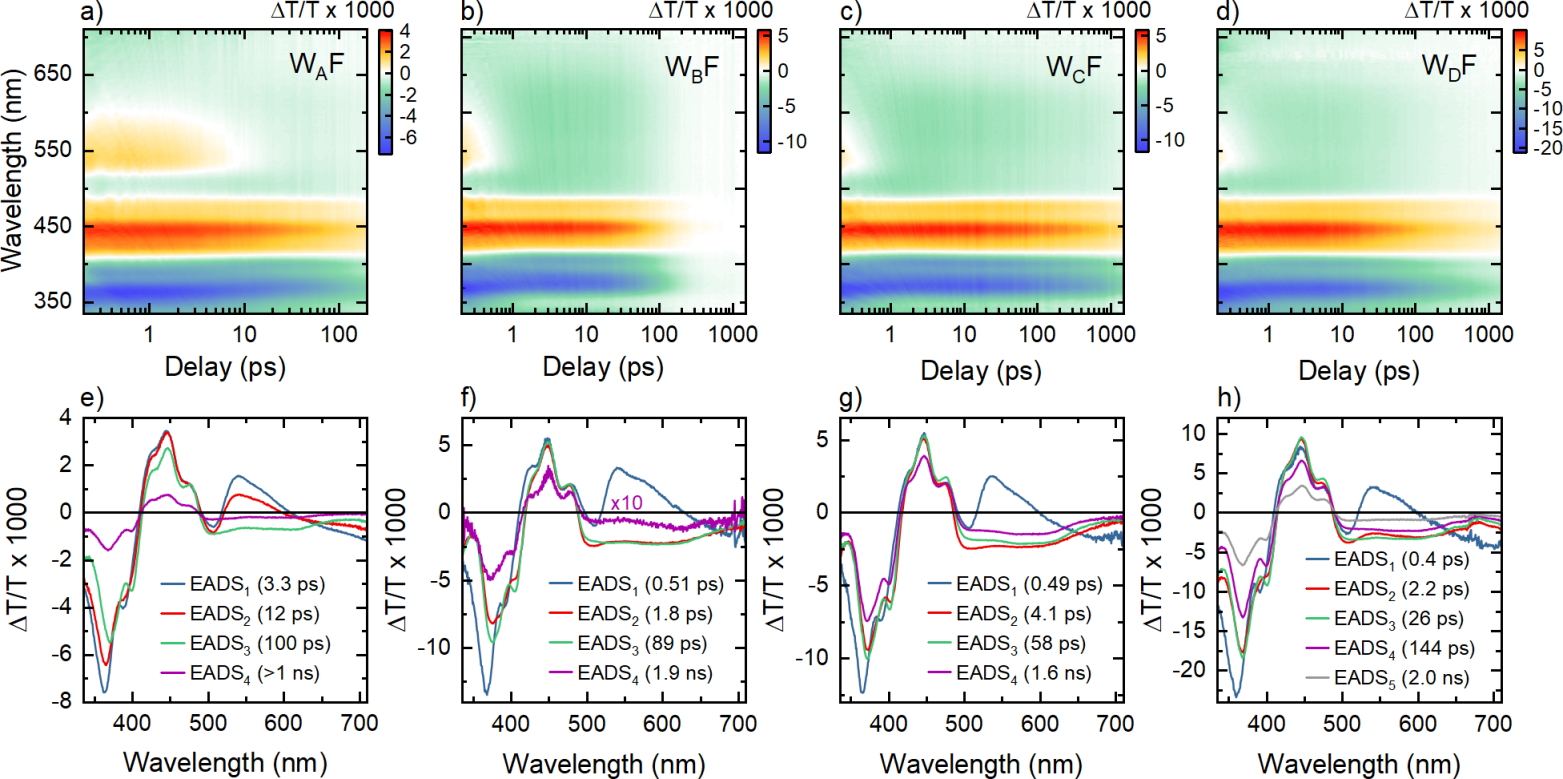
Transient absorption spectroscopy of *Er*Cry4a mutants. (a–d) Differential transmission Δ*T*/*T* spectra of the mutants W_A_F to W_D_F for delays between 0.2 ps and 1.5 ns (logarithmic time axis). (e–h) Corresponding evolution-associated difference spectra (EADS) for each mutant resulting from a global data analysis. (e) W_A_F mutant does not show a sub-picosecond decay component because the electron transfer from Trp_A_ to FAD_ox_ is suppressed by the removal of Trp_A_. (f–h) 0.5 ps decay components in the EADS spectra of W_B_F – W_D_F reflect this primary electron transfer from Trp_A_ to FAD. Each increase in the distance of the Phe mutation from the chromophore (W_B_F → W_C_F → W_D_F) results in one additional decay component in the EADS spectra, giving evidence for the second and third electron transfer steps in the electron transfer chain in *Er*Cry4a. A 2–4 ps decay component in all four mutants is assigned to vibrational cooling of the photochemically formed radical pairs. The slowest component for all four mutants corresponds to radical pair recombination on a nanosecond time scale.

Differential transmission (Δ*T*/*T*) spectra of the four Trp → Phe *Er*Cry4a mutants were recorded under comparable experimental conditions at a temperature of 1 °C in buffer solution at pH 8. Linearly polarized pump pulses with 30-fs duration centered at 450 nm were used to excite the π_2_ → π_3_ transition of the FAD chromophore within the proteins. Pump-induced changes in the transmission of the sample were probed using a broadband white-light continuum. The magic angle Δ*T*/*T* spectra are shown in Figure 2a–d for the four mutants for pump–probe delay times, *t*_W_, of up to 1.5 ns. All datasets were subjected to a global data analysis and can readily be explained using a multi-exponential decay model, including a minimal set of decay-associated difference spectra (DADS).^49^ Each DADS spectrum (Figure S7) describes a component of Δ*T*/*T* that decays or rises with an associated time constant. As such spectra are sometimes somewhat difficult to interpret, we have also calculated evolution-associated difference spectra (EADS;^49^ see Methods). Here, EADS_1_ represents the Δ*T*/*T* spectrum before the onset of the first of the sequential incoherent relaxation steps, while in simplified terms, EADS_*n*_ (*n* > 1) is the Δ*T*/*T* spectrum after the (*n* – 1)th relaxation process has been completed. The EADS spectra are presented in Figure 2e–h.

#### 2.2.1 Transient Absorption of *Er*Cry4a W_B_F

**Figure 3:**
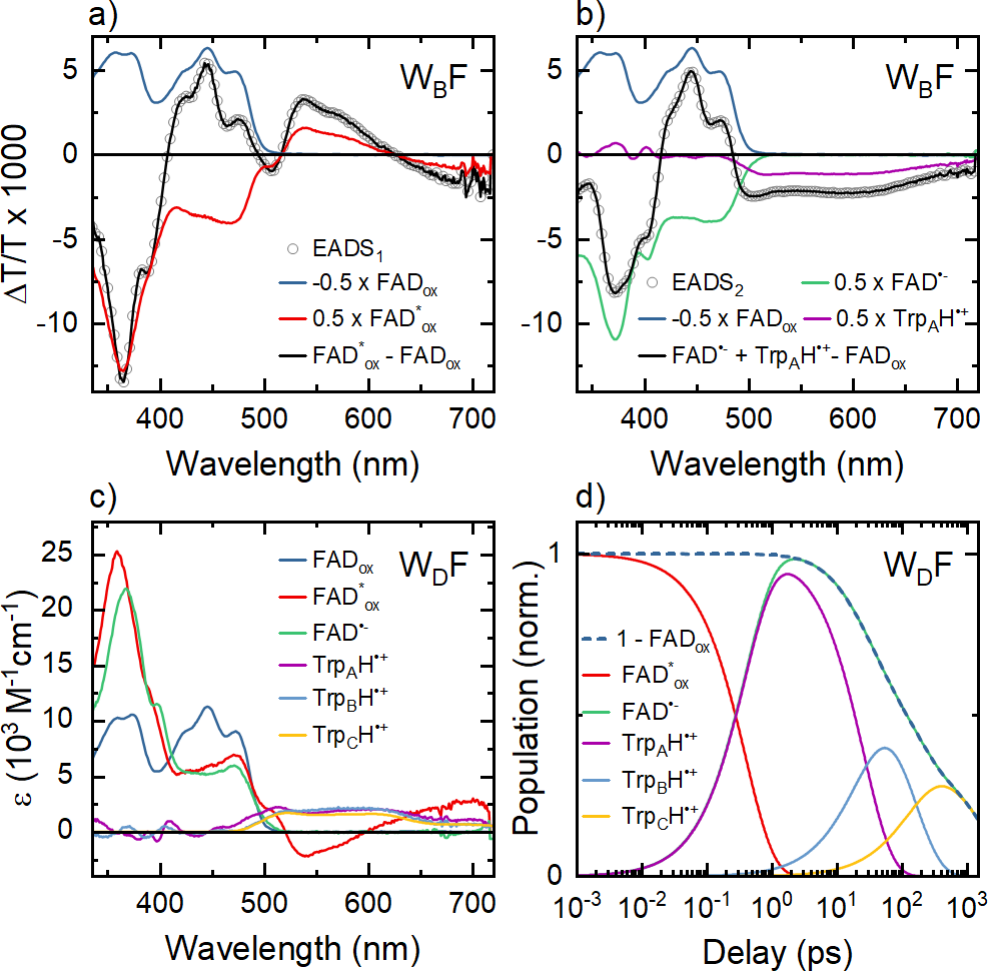
Analysis of the differential transmission, Δ*T*/*T*, spectra of *Er*Cry4a mutants. (a) EADS_1_ spectrum of W_B_F with 0.51 ps decay time (open circles) and its decomposition into the Δ*T*/*T* spectra of FAD_ox_ and FAD_ox_^*^ (solid lines). (b) EADS_2_ spectrum of W_B_F with a 1.8 ps decay time (open circles) and its decomposition into the Δ*T*/*T* spectra of FAD_ox_, FAD^•–^, and Trp_A_H^•+^ (solid lines). (c) Differential transmission Δ*T*/*T* spectra of the various neutral and charged radicals in *Er*Cry4a as deduced from the analysis of W_D_F. (d) Population dynamics of the neutral and charged radical states in W_D_F.

We first consider the W_B_F mutant (Figure 2b,f), where the conceptually simplest, sub-picosecond electron transfer dynamics from Trp_A_ to FAD are expected. The distinct signature of this electron transfer is the disappearance of the stimulated emission (SE) band around 550 nm, as shown in Figure 2b on a 0.5 ps time scale. The Δ*T*/*T* spectra in Figure 2b at early time delays, represented by the first EADS spectrum (EADS_1_) in Figure 2f, were recorded before this electron transfer sets in. These Δ*T*/*T* spectra thus show pump-induced changes in the spectrum of only the oxidized species, FAD_ox_. The spectra are therefore not yet affected by the formation of radicals in the sample. Indeed, EADS_1_ (blue line in Figure 2f) can be understood on the basis of the known optical transitions of FAD_ox_ (Figure 3a). The pump excitation promotes electrons to vibrationally excited states of the π_3_ electronic state, giving rise to a positive (Δ*T* > 0) ground state bleaching (GSB) contribution to the differential transmission with a spectrum matching the absorption of FAD_ox_ (blue line in Figure 3a). The pronounced vibronic structure around 450 nm is evident in EADS_1_ and is a clear marker for optically excited FAD_ox_^*^.^29^ Rapid vibrational relaxation within π_3_, complete within ≤ 200 fs, gives rise to a strongly red-shifted SE (Δ*T* > 0) band (π_3_ → π_2_) around 550 nm (see the red line in Figures 3a and S8). The GSB below 400 nm is obscured by a pronounced excited state absorption (ESA, Δ*T* < 0) centered at 360 nm (with a weak fine structure around 390 nm) from the π_3_ → π_5_ transition^47^ (red lines in Figure 3a,c). In addition, the Δ*T*/*T* spectra suggest broadband ESA of FAD_ox_^*^, spanning the range from 500 to 700 nm and a spectrally almost flat ESA between 400 and 470 nm that is more difficult to assign because the absorption of FAD_ox_^*^ has yet to be modeled with high accuracy (red lines in Figure 3a,c). Similar experimental ESA spectra have also been recorded by Kutta et al. but were not well-reproduced by their quantum chemical calculations.^29^

Importantly, the overall shape of EADS_1_ in Figure 2e–h is very similar in all four *Er*Cry4a mutants and the relative amplitudes of the different contributions remain basically unchanged. Since mutation alters the Trp chain with little effect on the FAD chromophore, this provides further support for the assignment of EADS_1_ to the Δ*T*/*T* spectrum of the FAD chromophore in its oxidized form. The most obvious dynamic feature of the Δ*T*/*T* map for the W_B_F mutant (Figure 2b) is the complete disappearance of the π_3_ → π_2_ SE band around 550 nm (decay time, 0.5 ps), while the amplitude of the GSB bands remain unchanged. This indicates that the decay of the excited state π_3_ population is not due to the refilling of the FAD_ox_ ground state orbital, π_2_, but reflects the formation of the negatively charged FAD^·–^ radical, most likely due to the transfer of an electron from Trp_A_. The map in Figure 2b also shows more subtle features. After the first electron transfer has been completed, the Δ*T*/*T* spectrum, now given by EADS_2_ in Figure 2f, has changed substantially. Not only has the characteristic SE band around 550 nm disappeared completely, but also the sharp peak in the FAD_ox_^*^ absorption around 360 nm and the broad ESA tail (red line in Figure 2f) have vanished in EADS_2_. While the GSB and ESA around 450 nm do not change when switching from EADS_1_ to EADS_2_, a new narrow ESA band around 370 nm with a side peak at 400 nm and a broad ESA band in the 500–700 nm range emerge in EADS_2_. After subtracting the GSB contribution from EADS_2_, a Δ*T*/*T* spectrum is obtained that quantitatively matches the sum of the absorption spectra of the FAD^•–^ radical anion and the TrpH^•+^ radical cation known from the literature.^29,45^ The corresponding spectra deduced from EADS_2_ are shown as solid lines in Figure 3b.The signatures of the spectrum of TrpH^•+^ (purple line in Figure 3c) are the broad absorption centered around 560 nm and the tail of a UV absorption band at 335 nm at the edge of the probe window.^29,39,50^ Theoretical studies of the FAD^•–^ spectrum show a transition from the singly occupied π_3_ to the unoccupied π_5_ orbital,^47^ experimentally seen in the peak in Figure 3b (circles) at 370 nm, red-shifted by ca. 10 nm with respect to the corresponding transition in FAD_ox_. A weak peak at 400 nm in EADS_2_ is assigned^47^ to the transition π_1_ → π_3_ and a broad resonance between 400 and 470 nm (green line in Figure 3b) is assigned to π_2_ → π_3_. The decomposed spectra agree well with those reported for other types of cryptochrome, suggesting that the disappearance of FAD_ox_^*^ leads to the formation of the [FAD^•–^Trp_A_H^•+^] radical pair. The associated decay time of τ_1_ = 1/*k*_1_= 0.51 ps is therefore the time constant for the first electron transfer in *Er*Cry4a activation, corresponding to the [FAD_ox_^*^ Trp_A_H] _→_^*k*_1_^ [FAD^•–^ Trp_A_H^•+^] process. Since the characteristic markers for FAD_ox_^*^, i.e., the SE emission band around 550 nm and the ESA peak around 360 nm, are completely absent in EADS_2_, we conclude that the yield for this first electron transfer, η_1_, is close to unity.

Our data analysis reveals some dynamics associated with a second time constant, τ_v_ = 1.8 ps. This process, however, has only a minor effect on the Δ*T*/*T* spectra (Figure 2f). Close inspection reveals a slight narrowing of the TrpH^•+^ band around 560 nm and a minor reshaping of the FAD^•–^ peaks at 370 and 400 nm. This has recently been associated with vibrational cooling of the optically formed radicals^29^ or, alternatively, to a second electron transfer along the Trp chain.^45^ The residual Δ*T*/*T* signal decays almost entirely with a time constant of τ_r_ = 89 ps. This is the decay of the photo-induced radical pairs by geminate recombination which refills the ground state, FAD_ox_. The residual, weak EADS_4_ spectrum in Figure 2f, with a shape matching that of EADS_3_, but with 20-fold smaller amplitude, suggests that a small concentration of radical pairs still exists for *t*_W_ ≫ τ_r_. Importantly, the Δ*T*/*T* spectra of W_B_F show no clear signatures of other electron transfer processes except for *k*_1_.

#### 2.2.2 Transient Absorption of *Er*Cry4a W_A_F

The substitution of Trp_A_ by Phe in the W_A_F mutant is expected to block the first electron transfer. Initially, the Δ*T*/*T* spectra in Figure 2a (EADS_1_, blue line in Figure 2e) are very similar to those of W_B_F. The fast dynamics on the sub-picosecond time scale, however, are no longer observed, which directly confirms the assignment of the first electron transfer in the dynamics of W_B_F. The fastest electron transfer dynamics in W_A_F occur with a time constant of 3.3 ps and result (EADS_2_, red line in Figure 2e) in a partial reduction of the SE and ESA signals of FAD_ox_^*^. Since electron transfer processes involving Trp_A_ cannot be the origin of this decay, the observed dynamics are most likely due to the FAD. Indeed, photo-induced electron transfer from the adenine to the flavin group in the FAD cofactor is known to occur in solution on a 5 ps time scale for the stacked conformation of the two aromatic rings.^27,31^ Although the exact time scale for the back electron transfer from flavin to adenine is somewhat uncertain for free FAD in solution,^27,31^ it is generally thought to happen on a time scale similar to the forward electron transfer, giving rise to an equilibrium between neutral and charge-separated states. The existence of such an equilibrium for the intra-FAD electron transfer in the W_A_F mutant may be the reason why the recorded data show only a partial decay of FAD_ox_^*^ with a 3.3 ps time constant. More surprising are the Δ*T*/*T* dynamics with a larger, 12 ps, time constant. After its completion, the resulting EADS_3_ (green line in Figure 2a) matches that seen in the W_B_F mutant, indicating the formation of a [FAD^•–^Trp_X_H^•+^] radical pair. MD simulations of the protein structure (Figure S9a) indicate that two additional Trp residues, Trp290 and Trp350, are present at edge-to-edge distances of 4 Å from the adenine and 7.5 Å from the flavin, respectively. These residues could potentially be involved in the formation of a [FAD^•–^TrpH^•+^] radical pair. The Δ*T*/*T* changes in Figure 2a disappear almost completely on a 100 ps time scale, and we associate this time constant with an effective geminate recombination of the [FAD^•–^TrpH^•+^] radical pair. The experiments prove the absence of fast electron transfer with sub-picosecond time constant in the W_A_F mutant.

#### 2.2.3 Transient Absorption of *Er*Cry4a W_C_F and W_D_F

The above analysis puts us in an excellent position to explore the electron transfer along the Trp chain in the *Er*Cry4a W_C_F and W_D_F mutants (Figure 2c,d). In both cases, the Δ*T*/*T* spectra at early time delays (EADS_1_ in Figure 2g,h) are very similar to those discussed before for the W_A_F and W_B_F mutants. In the case of W_C_F and W_D_F, the initial dynamics point to a first electron transfer from Trp_A_ to FAD with close to 100% efficiency and time constants of τ_1_ = 0.49 ps (W_C_F) and 0.4 ps (W_D_F), respectively. Also, the rapid decay of EADS_2_, seen for the W_B_F mutant, occurs with time constants of τ_v_ = 4.1 ps (W_C_F) and 2.2 ps (W_D_F). In contrast to the W_B_F case, the shape of the spectrum of the Trp radical cation changes slightly when going from EADS_2_ to EADS_3_, most obviously around 500 nm and in the far-red region. Since EADS_2_ is not affected by an extension of the Trp chain, our data do not support the assignment of τ_2_ to an electron transfer process. Instead, this observation presents strong arguments in favor of vibrational cooling as suggested earlier.^29^ The spectra recorded for both W_C_F and W_D_F mutants show a third EADS_3_ component with a spectral lineshape that is very similar to that observed for the W_B_F mutant. The EADS_3_ spectra decay with characteristic times of τ_2_ = 58 ps for W_C_F and 26 ps for W_D_F, respectively. In stark contrast to W_B_F, however, a fourth EADS_4_ component now appears after the third relaxation step with an amplitude approximately 2/3 that of EADS_3_, and with an almost identical lineshape. This finding implies that even after the third relaxation step, i.e. after about 100 ps, the excited *Er*Cry4a still contains a sizeable concentration of radical pairs and that geminate recombination is not yet complete. This observation thus points to a secondary electron transfer step, [FAD^•–^Trp_A_H^•+^] _→_^*k*_2_^ [FAD^•–^Trp_B_H^•+^], in which an electron is transferred from Trp_B_ to Trp_A_ with a rate constant *k*_2_ = 1/τ_2_. The data in Figure 2g,h indicate that this secondary electron transfer occurs with a yield of η_2_ ≃ 0.77 for W_C_F and 0.71 for W_D_F. In the case of W_C_F, the resulting radical pairs then recombine very slowly with τ_r_ ≃ 1.6 ns. In W_D_F, both the GSB and the radical pair absorption decay more rapidly than for the other mutants. The analysis in Figure 2h reveals the presence of a new decay channel with a time constant τ_3_ = 144 ps. This relaxation process has little effect on the shape of the Δ*T*/*T* spectra: the spectra EADS_3_, EADS_4_ and EADS_5_ differ only in amplitude. We assign the observed decay to the third electron transfer step, [FAD^•–^Trp_B_H^•+^] _→_^*k*_3_^ [FAD^•–^Trp_C_H^•+^], in which an electron moves from Trp_C_ to Trp_B_ with a rate constant *k*_3_ = 1/τ_3_ and a yield η_3_ ≃ 0.52. This yield is significantly lower than that of the second step. Figure 3d summarizes the population dynamics of the different neutral and charged radical states in W_D_F predicted by the analysis of the Δ*T*/*T* spectra. Only a small fraction, about 20–25%, of the initially generated radical pairs is still present after the first three electron transfers along the Trp chain (yellow line in Figure 3d). These radical pairs then undergo geminate recombination on a time scale τ_r_ = 2.0 ns (EADS_5_). Comparative studies of several samples of the same mutant indicate that the number of decay components needed to explain the experimental data does not depend on the specific sample studied. Also, the decay times and yields are quite similar.

#### 2.2.4 Transient Absorption of Wild Type *Er*Cry4a

**Figure 4:**
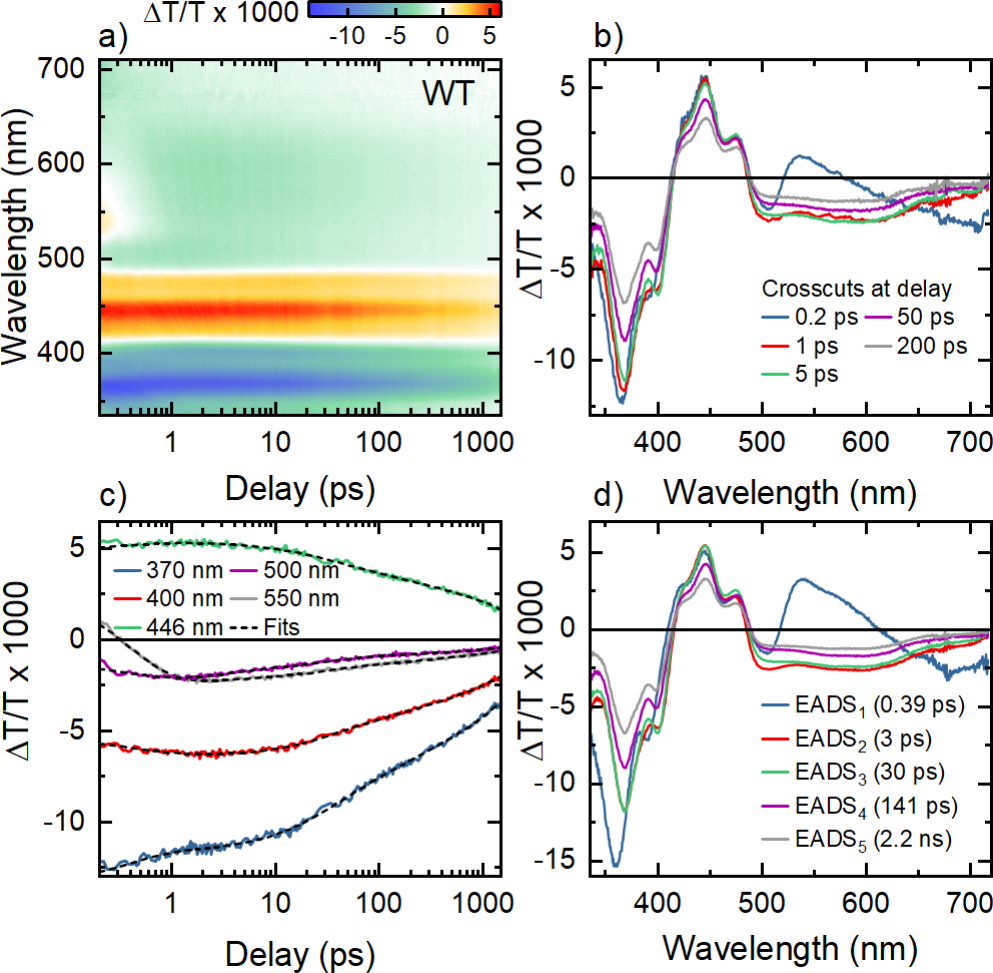
Transient absorption spectroscopy of wild type *Er*Cry4a. (a) Differential transmission Δ*T*/*T* spectra of the wild type protein for delays been 0.2 ps and 1.5 ns (logarithmic time axis). (b) Δ*T*/*T* spectra at selected delays. (c) Dynamics of the Δ*T*/*T* spectra at selected wavelengths. The results of the global analysis are shown as black dashed lines. (d) EADS spectra as obtained from the global data analysis. The resulting spectra are very similar to those of the W_D_F mutant. Based on the analysis of the mutant spectra, we assign the components with decay times of 0.39, 30, and 141 ps to the first, second, and third sequential electron transfer along the tryptophan chain of the protein. The 3 ps decay component is associated with vibrational cooling of the optically generated radical pairs, while the 2.2 ns component reflects radical pair recombination.

We now compare the results in Figure 2 to those for wild type *Er*Cry4a studied under the same experimental conditions. Qualitatively, the Δ*T*/*T* spectra in Figure 4a look almost identical to those recorded for the W_D_F mutant. Spectra at selected *t*_W_ are shown in Figure 4b and the dynamics at different probe wavelengths are compared to the results of a global fit analysis in Figure 4c. As in the case of the W_D_F mutant, this analysis reveals five distinct EADS spectra with features that closely resemble those of the W_D_F mutant. Also, the decay times associated with those five components are almost the same as for W_D_F. This strongly indicates that the sequential electron transfer model developed from the spectra of the mutants can be used to explain the wild type data. As such, we assign the time constant τ_v_ = 3 ps to vibrational cooling. The decay time of EADS_5_ of τ_r_ = 2.2 ns is identified with a geminate recombination process.

Our experimental results provide clear evidence for three sequential electron transfers in wild type *Er*Cry4a, with decay constants *k*_1_ = 1/0.39 ps^–1^, *k*_2_ = 1/30 ps^–1^, and *k*_3_ = 1/141 ps^–1^. The associated yields of the individual steps are η_1_ = 1.0, η_2_ = 0.78, and η_3_ = 0.77. In these measurements, the yield of the third step is somewhat higher than that for the W_D_F mutant. Thus, the concentration of radical pairs for the wild type protein is more than half the initial concentration of FAD_ox_^*^. The electron transfer rate constants and quantum yields deduced for the wild type protein and all mutants are summarized in Table 1. Importantly, our analysis of the transient absorption spectra of all four mutants gives no clear indication of back electron transfers within the FAD-Trp chain. All transients, except for the geminate recombination (τ_r_) process, can be well understood using a simple rate equation model considering only the forward steps. We therefore conclude that the back transfers are much slower than the forward steps and in fact too slow to be revealed by our experiments.

**Table 1:** Experimentally Obtained Electron Transfer Time Constants and Yields for Mutant and Wild Type Forms of *Er*Cry4a

protein	ET_1_ (η_1_)	ET_2_ (η_2_)	ET_3_ (η_3_)
W_B_F	0.51 ps (100%)		
W_C_F	0.49 ps (100%)	58 ps (77%)	
W_D_F	0.40 ps (100%)	26 ps (71%)	144 ps (52%)
WT	0.39 ps (100%)	30 ps (78%)	141 ps (77%)

### 2.3 Quantum Mechanical/Molecular Mechanical Simulations

#### 2.3.1 Occupancy of Tryptophan Sites

Our measured electron transfer time constants can now be compared to theoretical predictions from real-time quantum mechanical/molecular mechanical (QM/MM) simulations.^41,51^ The entire cryptochrome molecule is too large for a purely quantum-mechanical treatment, and therefore hybrid methods must be employed. Our simulations were performed for wild type *Er*Cry4a to provide additional atomic-level insights into the nature of the charge separation process. This is the first attempt to carry out quantum simulations of electron transfer in *Er*Cry4a for direct comparison with experimental measurements. Earlier phenomenological studies^10^ helped to estimate the kinetics of the charge transfer steps but did not take explicit account of the atomistic dynamics of the protein environment.

The electron transfer times within the Trp-tetrad are ca. 100 ps or faster (Table 1). A non-adiabatic, semi-classical electron transfer scheme based on a fragment-molecular-orbital implementation of the semi-empirical density-functional method DFTB^41,51−54^ is therefore suitable for computational modeling. For an accurate description of the electron transfer process, it is vital to take account of the motion of the biological scaffold of the protein. Moreover, this essentially classical motion should be coupled to the quantum dynamics of the transferring electron. The DFTB approach has been used previously to study real-time electron transfers in frog and plant cryptochromes^41,51^ and is now applied here to model the ET_2–4_ steps (see Figure 1a) in *Er*Cry4a. The *Er*Cry4a structure required for this study was obtained by homology modeling, equilibrated using extended MD simulations, and validated by several prior analyses.^10,55,56^ The starting point for simulating the ET_2–4_ steps is the [FAD^•–^Trp_A_H^•+^] radical pair, produced by the first electron transfer, ET_1_. Here, an electron vacancy (hole) resides on the Trp_A_ residue and starts to propagate along the tryptophan chain, facilitated by the motion of the protein.^41^ As the hole progresses along the tetrad, one can calculate the probability that it is located on each of the tryptophans (the occupancies) and hence determine the electron transfer kinetics from their time dependence. Note that ET_1_ was not considered in our computational study because this step, which involves excited flavin and tryptophan, requires a special treatment within DFTB, while ET_2–4_ involve only tryptophan residues. See the Methods section for a description of the DFTB method.

**Figure 5:**
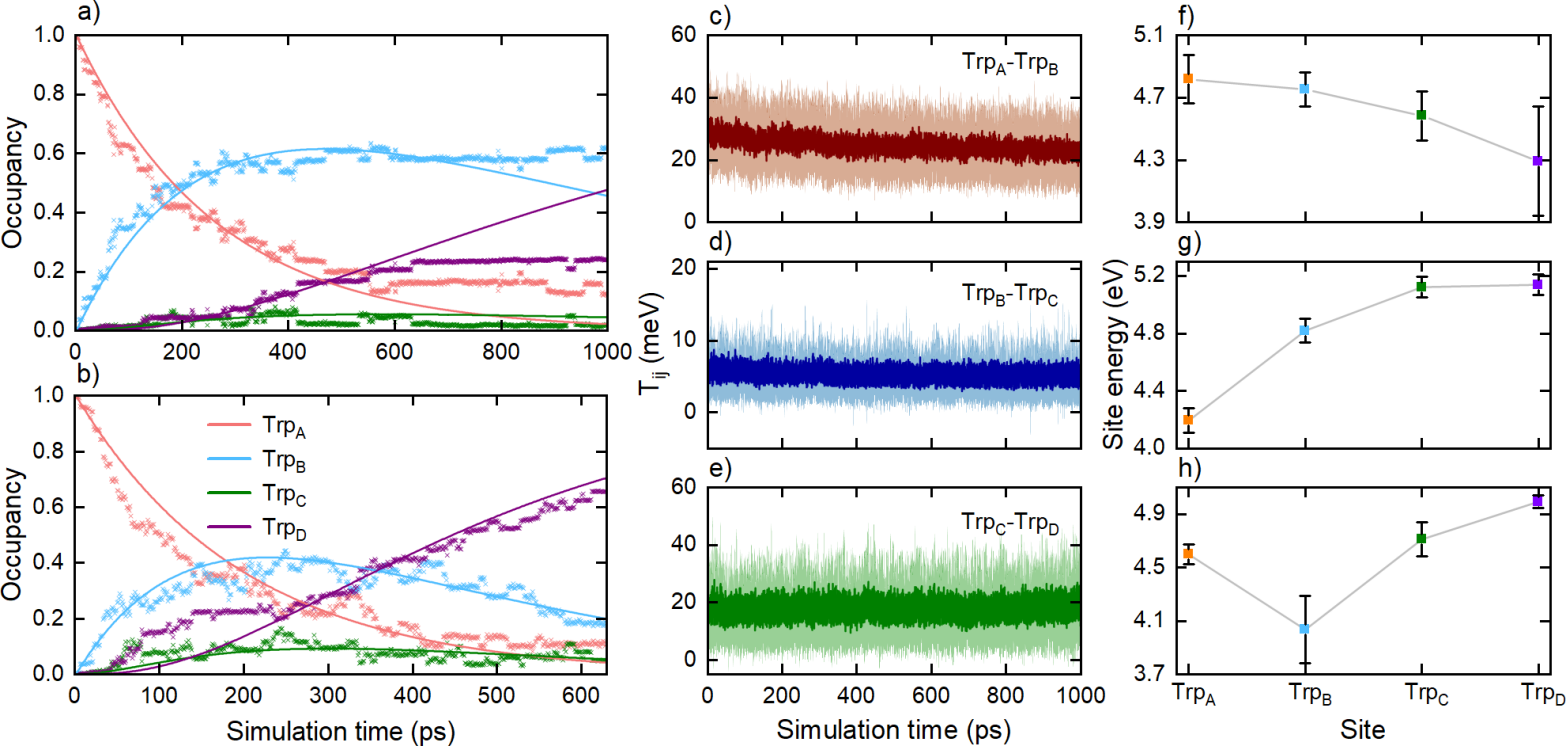
Characterization of electron transfers (ET_2_-ET_4_) in the Trp-tetrad by QM/MM DFTB simulations. (a) Average occupancy of the four tryptophan sites computed over 50 simulations (points), fitted using eq 1 (lines). (b) Average occupancy of the four tryptophan sites (points) computed over 27 simulations in which electron transfer is considered complete, i.e., the occupancy of Trp_C_H^•+^ or Trp_D_H^•+^ exceeds 0.3. The solid lines show a fit to eq 1. (c–e) Average electronic couplings computed over 50 simulations between two neighboring tryptophan sites as a function of simulation time (dark color) with the corresponding standard deviation (light color). (f–h) Average site energies of the different tryptophans divided into three electron transfer scenarios observed in the simulations: (f) average site energies for the completed electron transfers (*N* = 27); see Video S1; (g) average site energies for the transfers that do not go beyond the first tryptophan Trp_A_ (*N* = 13); see Video S2; and (h) average site energies for the transfers that stop at Trp_B_ (*N* = 60); see Video S3.

The time dependence of the occupancies of the four tryptophan sites is shown in Figure 5a (averaged over 50 1-ns simulations) and Figure 5b (averaged over 27 630-ps simulations). By 630 ps, the electron hole had reached Trp_C_H^•+^ or Trp_D_H^•+^ with a probability of at least 0.3. Figure 5a includes the results of all trajectories, independent of whether the electron transfer reached the end of the Trp-tetrad. The solid lines in Figure 5a,b correspond to the numerical fits to the simulated data, which were obtained from the solution of the set of coupled rate equations
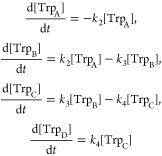
1Here, [Trp_*m*_] denotes the occupancy of the *m*-th tryptophan site and *k_n_* is the rate constant for the *n*-th electron transfer. This analysis gives estimates of the characteristic times of each electron transfer step; the results are summarized in Table 2. Based on our experimental results, we assume that all back transfer steps in the rate equation model are much slower than the corresponding forward rates and can therefore be neglected. Given the limited number of simulation runs currently available and the resulting statistical uncertainty, we cannot exclude the existence of small contributions from back electron transfer.

**Table 2:** Estimated Electron Transfer Times Obtained from Simulations of Electron Propagation along the Trp-Tetrad^a^

dataset	ET_2_ (ps)	ET_3_ (ps)	ET_4_ (ps)
1 ns (Figure 5a)	260	960	90
630 ps (Figure 5b)	200	260	60

a Results are shown for the numerical fits of the solution of eq 1 to the computed data for the 1-ns simulation and 630-ps simulations, averaged over 50 and 27 independent simulations, respectively. In the latter case only those simulations where Trp_C_H^·+^ or Trp_D_H^·+^ obtained an occupancy of at least 0.3 were considered. The values have been rounded to the nearest 10 ps.

This analysis demonstrates the possibility of electron transfer between the tryptophans in *Er*Cry4a. However, Figure 5a shows some discrepancies between the simulated data and the fitted curves at longer times—differences that are less pronounced in Figure 5b. The poor fitting of the simulation data in Figure 5a is due to the large uncertainties in the average occupancy values of the four Trp_A–D_ sites. In a significant fraction of the simulations, electron transfer was not complete within the 1-ns time span. In a subset of the trajectories, the hole in Trp_A_ rarely jumps to Trp_B_. This is a natural effect which arises because different initial structures support the ET process to different extents, and some do so rather inefficiently. A much larger number of simulations, associated with a prohibitive computational effort, would be required to take a proper account of this dependence. The current protocol is a practical solution that results in relatively minor overestimates of the rate constants of the individual ET steps. To illustrate the influence on the overall kinetics of those simulations in which the hole does not get as far as the end of the Trp-chain, we have included in Figure 5b data only for those simulations in which the occupancy of Trp_C_H^•+^ or Trp_D_H^•+^ reaches at least 0.3 by the end of the simulation. This gives a much better correspondence between the average occupancies of the tryptophans and the solutions of eq 1. The results in Figure 5b show the electron transfers Trp_B_H → Trp_A_H^•+^, followed by Trp_C_H → Trp_B_H^•+^, both on a 200 ps time scale. The fourth electron transfer, Trp_D_H → Trp_C_H^•+^, is predicted to be somewhat faster (<100 ps). Thus, the occupancy of Trp_C_H^•+^ will always be low, making it difficult to distinguish the occupancies of Trp_C_H^•+^ and Trp_D_H^•+^ in an experiment. This agrees well with our experimental observations for the wild type and mutant proteins. The electron transfer times derived from the simulations are presented in Table 2.

#### 2.3.2 Marcus Theory Analysis

The results of the DFTB simulations can be qualitatively understood if we consider weak coupling between the donor and acceptor sites and assume the high temperature limit. In this case, the electron transfer rate constant, *k*_ET_, can be estimated using Marcus theory,^57^ as:
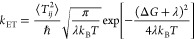
2*k*_ET_ is governed by three parameters: the coupling between the donor and acceptor, *T_ij_* (*ij* = AB, BC, CD); the thermodynamic driving force, Δ*G*; and the reorganization energy λ. The driving force is related to the site energies.^41^ The electronic coupling between two tryptophan sites is defined as *T_ij_* = ⟨ϕ_*i*_|*Ĥ*_0_|ϕ_*j*_⟩, while the site energies can be identified as the ionization potentials of the respective tryptophans as ϵ_*i*_ = ⟨ϕ_*i*_|*Ĥ*_0_|ϕ_*i*_⟩. Here, ϕ_*i*_ denotes the molecular orbitals of the electrons localized on the respective tryptophan residues, while *Ĥ*_0_ is the Hamiltonian of the system, including contributions from the solvent. Thus, Coulomb corrections to Δ*G*^58^ are intrinsically included in the DFTB simulations and, in fact, create a major part of the driving force for sequential electron transfer.^41^

Figure 5c–e shows the time evolution of the electronic couplings *T_ij_* of adjacent tryptophans. These interactions are the fundamental results of the DFTB calculations and have been extensively discussed previously.^41,51^ In particular, it has been shown that despite the simplicity of the DFTB approach, it yields accurate coupling values for charge transfer between tryptophan residues in cryptochromes and photolyases,^41,51,59^ comparable with values obtained using higher order methods. Physically, the electronic couplings determine the probability of electron transfer between the sites (eq 2). The average coupling of Trp_B_ and Trp_C_ (*T*_BC_) is around 5 meV, while that of Trp_C_ and Trp_D_ is significantly higher, at 20–30 meV.

The average site energies of the four tryptophans are shown in Figure 5f–h for three cases: simulations containing completed electron transfers (Figure 5f and Video S1), simulations with no electron transfer from Trp_B_H to Trp_A_H^•+^ (Figure 5g and Video S2), and simulations with no electron transfer from Trp_C_H to Trp_B_H^•+^ (Figure 5h and Video S3). The three outcomes correspond to very different site energies. Note that while the electronic couplings *T_ij_* contribute quadratically to the overall electron transfer rate constant (see eq 2), Δ*G* and λ (which are determined by the site energies) contribute exponentially and so may have more of an effect on *k*_ET_. The average site energy difference between Trp_A_ and Trp_B_ is −0.05 eV for the cases with completed charge transfers; +0.62 eV when there is no transfer from Trp_B_H to Trp_A_H^•+^; and −0.56 eV for the case where the hole gets stuck at Trp_B_H^•+^. The average difference between Trp_B_ and Trp_C_ is +0.68 eV for the simulations in which the Trp_C_H → Trp_B_H^•+^ step does not occur. Thus, in Figure 5f, ET is energetically downhill all the way to Trp_D_; in Figure 5g, it is uphill from the start; and in Figure 5h, the first step is downhill, but subsequent electron transfers are energetically unfavorable.

## 3 Discussion

The ability to express high purity Cry4a from night-migratory European robins and to replace the tryptophan residues individually by redox-inactive phenylalanines has allowed us to obtain new microscopic insight into the light-induced electron transfer dynamics along the Trp-tetrad chain that connects the flavin chromophore to the surface of the protein. Using ultrafast optical spectroscopy, in connection with a Marcus model for the electron transport inside the protein, we have unraveled several of the primary steps in the photocycle of this photoreceptor, which has been suggested to be crucial for the magnetic compass sense of migratory songbirds.^10,11,14^

When the tryptophan (Trp_A_) closest to the FAD chromophore is replaced by a phenylalanine, electron transfer along the Trp-tetrad is efficiently suppressed and slower electron transfer pathways are revealed, most likely involving tryptophan residues that are not part of the tetrad. In all the other Trp → Phe mutants we have studied and in the wild type protein, Trp_A_ serves as an efficient donor, transferring an electron to the FAD within 0.5 ps of photoexcitation with a quantum yield of unity. The experiments reveal mutation-insensitive vibrational cooling dynamics within the manifold of radical pair states on a timescale of few picoseconds. Thesy also provide evidence for a cascade of sequential electron transfers in which the second (Trp_B_ → Trp_A_) and third (Trp_C_ → Trp_B_) steps occur with transfer times of 30 and 140 ps, respectively, and can be selectively blocked by mutation.

The yields of the final step in generating the charge-separated states in the wild type protein and the W_D_F mutant are >75% and ∼50%, respectively. Our measurements, which probe the dynamics over the first 1.5 ns after photoexcitation, do not provide a clear indication for a fourth transfer step, from Trp_D_ to Trp_C_, in wild type *Er*Cry4a, either because of the difficulty of distinguishing these distant tryptophans or because this step is too fast or too slow to be resolved. A comparison of the transient absorption data from the wild type protein and the W_D_F mutant suggests that the presence of the fourth tryptophan in the chain has very little effect on the dynamics within the first 1.5 ns, beyond an increase in the concentration of radical pairs at the end of the measurement window. Such a change in concentration could be explained, for example, by the fourth electron transfer, from Trp_D_ → Trp_C_, being faster than the third and the formation of an equilibrium between the associated radical pairs.^10,18^ The present data indeed point to the formation of such an equilibrium and suggest more refined studies of the functional role of the terminal Trp.

While the time constants we have deduced for the first and second electron transfers agree well with those recently reported by Kutta et al. for fruit fly cryptochrome,^29^ the kinetics measured here are somewhat slower than recent predictions^10^ based on MD simulations and Moser–Dutton theory. They are also faster than those predicted by the DFTB-based QM/MM simulations presented here. In the QM/MM simulations, the first ET_1_ could not be modeled and will remain an open question until reliable DFTB parameters become available for the excited flavin chromophore. The simulations give atomistic insight into the sequential electron transfer cascade along the Trp-tetrad with typical transfer times in the 100 ps range. Interestingly, the simulations suggest that for certain *Er*Cry4a configurations, electron transfer does not proceed to the end of the tetrad but gets stuck at Trp_A_H^•+^. In such cases, the calculations suggest an increase in average site energy on going from Trp_A_H^•+^ to Trp_B_H^•+^ of 0.62 eV. In contrast, when this electron transfer does take place, it is because there is a decrease in the average site energy. The increases in site energy found for some of the simulations cannot be attributed to a single amino acid residue but are a collective effect of the protein environment that, overall, appears slightly different in the various starting configurations used in the QM/MM simulations.

The characteristic electron transfer times for the ET_2_ and ET_3_ processes obtained from the simulations in which the hole reaches the end of the Trp-tetrad agree, within an order-of-magnitude, with experiment. Comparing Tables 1 and 2, the agreement is better for ET_3_ than for ET_2_. Computationally, we could also establish a characteristic time for ET_4_ (60 ps), which has hitherto been inaccessible to experimental measurement. This step is somewhat faster than the preceding electron transfers, which would explain why it has been difficult to separate ET_3_ and ET_4_ experimentally. The theoretical electron transfer times follow from a simple kinetic model, eq 1, which was reasonably successful in describing the calculated average time dependence of the occupancies of the four tryptophan sites. The model did not include back electron transfer steps, which are expected to be significantly slower than the forward electron transfers as found in earlier studies^40,41,51^ and in the present measurements.

To minimize photodegradation of the light-sensitive proteins, our pump–probe experiments have all been performed at 1 °C and pH 8.0, under which conditions the samples are photostable for several hours. The conditions in a cell in a living bird are somewhat different: 40–43 °C and pH ∼7.3.^60,61^Thermodynamically, the main change in the charge states of proteins that results from a reduction in temperature is a shift in the acid–base equilibrium of histidine side chains, which can be reversed by an increase in pH. This suggests that, in terms of the protein structure, 1 °C and pH 8 should resemble the physiological conditions.^61,62^

Turning from thermodynamics to dynamics, the internal mobility of the protein will be strongly temperature-dependent. The effect this has on the electron transfer kinetics can be estimated from the Marcus model, as shown in eq 2, which suggests that the electron transfer times would decrease by only ca. 50% when the temperature is increased from 1 to 42 °C, the body temperature of a European robin. As such, we expect the main conclusions of the present work, which deals exclusively with the first nanosecond after photo-excitation, to remain valid. A preliminary study indeed suggests that the electron transfer rates and yields do not change greatly between 1 and 30 °C. This temperature dependence will be discussed in more detail in a forthcoming publication.

Finally, it should be noted that the magnetically sensitive steps in the photochemistry of cryptochromes all take place on timescales from hundreds of nanoseconds to seconds, minutes and possibly hours.^10^ Many of these processes are likely to be strongly temperature-dependent. Examples include the protonation and deprotonation of the flavin and tryptophan radical ions,^63^ electron spin relaxation,^17^ and the conformational changes that are necessary to form the state of the protein that initiates signal transduction.^11^ None of these processes is amenable to the ultrafast methods used here, and their temperature dependence will need to be investigated using other techniques.

## 4 Materials and Methods

### 4.1 Sample Preparation

Wild type *Er*Cry4a (GenBank: KX890129.1) and its four mutants W_X_F (X = A–D) were cloned, expressed, and purified according to the protocol described by Xu et al.^10^ Briefly, the tryptophan mutants were generated by replacing the DNA codon for tryptophan (TGG) at amino acid position 395 (W_A_F), 372 (W_B_F), 318 (W_C_F), or 369 (W_D_F) in the *Er*Cry4a gene by a phenylalanine codon (TTT) in a polymerase chain reaction using the Q5 site-directed mutagenesis kit (New England Biolabs). Plasmids were confirmed by Sanger sequencing (LGC Genomics). Proteins were expressed in BL21(DE3) *E. coli* cells in the dark and purified by Ni-NTA agarose columns, followed by anion exchange chromatography. Purified protein samples were concentrated to 5–6 mg/mL in an aqueous buffer solution (20 mM Tris, 250 mM NaCl, and 20% glycerol) along with 10 mM of the reducing agent 2-mercaptoethanol (BME) to avoid dimerization of the protein. Samples were snap-frozen in liquid nitrogen and stored at −80 °C for 3–5 days until the measurements were made. Since the photocycle starts from the fully oxidized state FAD_ox_, the reducing agent was removed and the sample was fully oxidized prior to the optical measurements. For this, the protein sample was washed with BME-free buffer solution in a Millipore centrifugation filter (Amicon Ultra, 30 kDa) using a temperature-controlled microcentrifuge (4 °C, 14,000 rpm). This step also removed free FAD from the sample. Addition of 1.5 mM potassium ferricyanide (PFC), followed by centrifugation for 1 h to remove aggregated proteins, was repeated until the sample was fully oxidized as confirmed by absorbance measurements. A remaining concentration of 1.7–2.6 mM PFC prevented photoreduction during the optical experiments. The PFC-corrected absorbance was used to determine the final concentration of the samples to be 120–220 μM using the molar extinction coefficient of FAD_ox_.^31^ All pump–probe experiments were performed at a temperature of 1 °C and in a buffer solution at pH 8.0 to avoid photodegradation of the light-sensitive protein. Transient absorption measurements on a buffer solution containing 1 mM PFC showed only a very weak nonlinear signal with a lifetime of a few picoseconds when pumped at 450 nm (see Figure S4), and no detectable PL was observed.

### 4.2 PL Experiments

PL measurements were performed using broadband (400–680 nm) 1.25 pJ pulses from a 40 MHz fiber laser source (SC400, Fianium). Emitted light was collected with a microscope objective with a numerical aperture of 0.3 and detected using a single-photon avalanche photodiode and a time-correlated single-photon counting unit (PicoHarp 300, PicoQuant). The instrument response time of the detection system at 550 nm is ∼60 ps. To obtain correlated excitation and emission spectra, a Fourier transform approach using two phase-stable common-path interferometers (Translating Wedge-based Identical pulse eNcoding System, TWINS^64^) was used,^65^ yielding a spectral resolution of ∼6 nm in excitation and emission wavelength. A more detailed description of the setup and data analysis can be found in the Supporting Information.

### 4.3 Transient Absorption Experiments

The measurements were performed using ∼30 fs pump pulses at 450 nm on a 15 μL volume of the sample in a quartz microcuvette (Hellma). The pulses are generated in an optical parametric amplifier (Topas, Light Conversion) pumped by regeneratively amplified 25-fs pulses at 800 nm (Legend Elite, Coherent) at a repetition rate of 10 kHz. Differential transmission spectra Δ*T*(λ, *t*_W_)/*T* = (*T*_on_(λ, *t*_W_) – *T*_off_(λ))/*T*_off_(λ) were recorded as a function of probe wavelength λ and time delay *t*_W_ using a broad supercontinuum probe, generated in a CaF_2_ crystal. Here, *T*_on/off_ denotes the probe transmission in the presence/absence of the pump, respectively. The spectra were recorded with parallel and crossed polarizations of the pump and probe beams. Scattering corrections to the Δ*T* spectra were made as described in the Supporting Information. The experiments were performed with 20 nJ pump pulses focused to a spot size of ∼50 μm, and it was ensured that the Δ*T*/*T* signals are well in the linear regime (see Figure S2). No change in signal due to sample degradation could be observed during the measurements (see Figure S3).

### 4.4 Data Analysis

Transient absorption scans recorded with parallel and crossed pump and probe polarizations were averaged, and isotropic, magic angle spectra were calculated. The few-ps chirp of the probe continuum was corrected by extracting the wavelength-dependent time delay zero (*t*_W_(λ) = 0) from the cross-phase modulation artifact of a transient absorption measurement of plain buffer solution. The first 200 fs of the corrected dynamics were discarded due to residual coherent signal contributions from the solvent. The datasets were subjected to a global analysis using a multi-exponential decay model.^66^ This decomposes the data into a set of *n* decay associated difference spectra (DADS_*i*_) with corresponding decay times τ_*i*_, Δ*T*/*T*(λ, *t*_W_) = ∑^n^_*i* = 1_DADS_*i*_(λ)e^–*t*_W_/τ_*i*_^. The lowest number of decays necessary to simultaneously reproduce the data at all wavelengths was taken. For more details, see the Supporting Information. The DADS spectra were then used to obtain evolution associated difference spectra EADS_*k*_(λ) = ∑^n^_*i* = *k*_DADS_*i*_(λ).

#### 4.4.1 MD Simulations

The structure of *Er*Cry4a in its dark state was adapted from earlier studies,^10,46,56^ where it was simulated in a 94 Å × 106 Å × 102 Å water box neutralized with 0.15 M NaCl, resulting in a total of 100,518 atoms. The structure was minimized for 500 conjugate gradient steps using a steepest descent minimization algorithm, followed by a second, 2 ns equilibration using the leap-frog integrator with a temperature of 300 K, kept constant by a Berendsen thermostat. The equilibration was followed by a 100 ns dynamic equilibration and 100 ns production simulation. Both equilibration and production simulations utilized a 2-fs timestep, and the LINCS algorithm^67^ was used to keep the lengths of bonds involving hydrogen atoms fixed at their equilibrium values. Periodic boundary conditions were adopted for all stages, and the particle-mesh-Ewald summation method was employed for evaluating the Coulomb forces. Van der Waals forces were calculated using a smooth cut-off of 12 Å with a switching distance of 10 Å. All calculations were carried out utilizing the GROMACS package.^52,68^ The MD calculation utilized the Amber99SB forcefields for proteins^69,70^ with earlier parametrized forcefields for the FAD cofactor.^41,51^

### 4.5 Non-adiabatic Simulation of Electron Transfer

The method for the real-time electron transfer has been carried out using a hybrid QM/MM DFTB protocol from two earlier studies on frog and plant cryptochromes.^41,51^ The protocol employed molecular fragments participating in the electron transfer as a defined segment of the system that was subjected to the quantum mechanical description (QM region), with the rest of the system being described using classical molecular mechanics force fields (MM region). The computational scheme utilizes a separation of frontier orbitals similar to the Hückel and Pariser-Parr-Pople models^71,72^ although in this case the orbitals were based on molecular fragments instead of atoms. For the DFTB calculations, a total of 100 structures (snapshots) were sampled from the production MD simulation, and each of the snapshots was taken at a 1-ns time interval. The QM region was for all QM/MM simulations selected as the four tryptophans. In this region, Trp_A_H^•+^ was assumed to have a missing electron, as the initial structure and FAD was assumed to have an extra electron, that is, being in its FAD^•–^ state. The snapshots were each simulated for 1 ns using a time step of 1 fs, though due to computational limitations, only 50 of the snapshots completed the 1 ns calculation, while the other 50 calculations resulted in a simulation length of 630 ps. The MM region was simulated using the Amber99SB forcefields for proteins^69,70^ with earlier parametrized forcefields for the FAD cofactor,^41,51^ while the QM region and electron transfer was estimated using earlier parameters.^73,74^

## Supporting Information Available

Additional experimental details, materials and methods, PL spectra, effect of pump pulse energy, stability of differential transmission spectra, reference measurements, DADS spectra, and spectral assignment (PDF)
Simulations for the completed electron transfer (AVI)
Simulations with no electron transfer from Trp_B_H to Trp_A_H^·+^ (AVI)
Simulations with no electron transfer from Trp_C_H to Trp_B_H^·+^ (AVI)

## Supplementary Material

ja3c00442_si_001.pdf

ja3c00442_si_002.avi

ja3c00442_si_003.avi

ja3c00442_si_004.avi
