# Supplementary material for: Tracking the Electron Transfer Cascade in European Robin Cryptochrome 4 Mutants
Source: J Am Chem Soc. 2023 May 17;145(21):11566–78. doi: 10.1021/jacs.3c00442 (PMC10236492; doi:10.1021/jacs.3c00442)
Supplement: Supplementary file 1 — ja3c00442_si_001.pdf [file ja3c00442_si_001.pdf]

Supporting Information for

Tracking the Electron Transfer Cascade in European Robin Cryptochrome 4 Mutants

Daniel Timmer[a], Anders Frederiksen[a], Daniel C. Lünemann[a], Anitta R. Thomas[a], Jingjing Xu[b], Rabea Bartölke[b], Jessica Schmidt[b], Tomáš Kubař[c], Antonietta De Sio[a,d], Ilia A. Solov'yov[a,d,e], Henrik Mouritsen[b,e] and Christoph Lienau[a,d,e]*

[a] Institut für Physik, Carl von Ossietzky Universität, 26129 Oldenburg, Germany
[b] Institut für Biologie und Umweltwissenschaften, Carl von Ossietzky Universität, 26129 Oldenburg, Germany

[c] Department for Theoretical Chemical Biology, Institute for Physical Chemistry, Karlsruhe Institute of Technology, 76131 Karlsruhe, Germany

[d] Center for Nanoscale Dynamics (CENAD), Carl von Ossietzky Universität Oldenburg, Institut für Physik, 26129 Oldenburg, Germany
[e] Research Centre for Neurosensory Science, Carl von Ossietzky Universität, 26111 Oldenburg, Germany

*Correspondence to: christoph.lienau@uni-oldenburg.de

Table of Contents

1. Photoluminescence excitation experiments 2
2. Transient absorption (TA) experiments 3
3. Linearity and sample stability 3
4. PFC oxidizer 5
5. FAD in buffer 5
6. Data preparation and analysis 6
7. Additional simulation data 9
8. SI references 10

1. Photoluminescence excitation experiments

Photoluminescence (PL) excitation experiments are performed by use of an interferometric time correlated single photon counting (TCSPC) setup (1). Here, a 40 MHz white light source (SC400, Fianium) is filtered to a spectral range of 400-680 nm with an average power of 50 µW. It passes a first, passively stable common-path interferometer (Translating Wedge-based Identical pulse eNcoding System, TWINS (2)) and is focused using a microscope objective (0.35 NA) into the sample. The sample solution is kept contained in a Hellma micro-cuvette at 1°C throughout the measurement. Directly before and after the sample a fraction of the incident/transmitted light is split off and measured with a photodiode to monitor the sample absorption over time. A second microscope objective (0.35 NA) placed at 90° with respect to the first one collects the light emitted from the sample that passes through a second TWINS interferometer and is finally focused onto a single-photon avalanche photodiode (PDM, Micro Photon Devices, 100x100 μm^2 sensor area). Both TWINS are scanned and a TCSPC histogram is recorded by the TCSPC electronics (PicoHarp 300, PicoQuant) at every position, additional to the photodiode signals.
The Fourier transform of the signal along both TWINS position axes yields excitation-emission spectra (3) at each TCSPC time point and the time-integrated excitation-emission spectrum, see Fig. S1a. The interferometric approach also allows us to separate out the coherent scattering contribution (1). A Matlab-based toolbox (4) is used to globally analyze the 3-dimensional dataset, decomposing it into independent sets of excitation and emission spectra and dynamics, see Fig. S1b-c and Fig. 1. The Fourier transform of the photodiode signals yields the spectrum of the exciting laser light and the absorption spectrum of the sample. The excitation-emission maps and excitation spectra are normalized to the white light spectrum to only feature sample properties. To compare the spectra of wild type *Er*Cry4 and the mutants with those of free FAD, a 40 µM solution of molecular FAD in a buffer that contains 1 mM PFC was measured. Excitation and emission spectra of all protein species are indistinguishable from those of free FAD_{ox} and no spectral signatures of protein-bound FAD redox states could be observed (Fig. S1).
This indicates that there are no measurable spectral signatures from protein-bound FAD in the excitation spectra of wild type *Er*Cry4 and its mutants and that essentially all of the emission stems from residual FAD molecules that are not bound to the protein. Only in mutant W_AF we see a faint emission from bound FAD_{ox}, more clearly resolved in the time-dependent measurements in Fig. 1g. We have deduced the concentrations of FAD chromophores in the protein samples and that of molecular FAD in solution from absorption measurements. By comparing the signal strength of the PL in the excitation-emission maps of the wild type proteins and the mutants to that of molecular FAD in buffer solution, we can then estimate the fraction of bound FAD. In all as-prepared samples, the fraction of bound FAD is at least 97%.

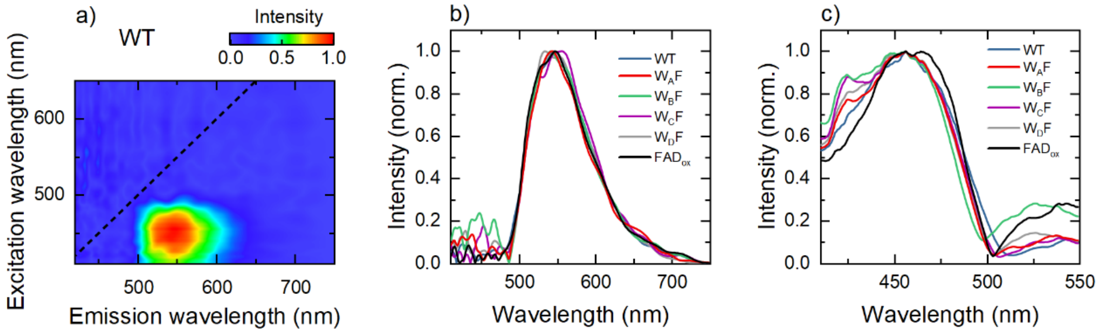

Figure S1: PL spectra of wild type *Er*Cry4 and mutants. a) Excitation-emission map of wild type *Er*Cry4 showing the excitation of a small amount of free FAD that is not bound to the protein. The emission from protein-bound FAD_{ox} is too weak to be seen in this map. b) Emission and c) excitation spectra extracted from the PL maps of wild type *Er*Cry4 (blue lines) and mutants. The lack of a vibronic substructure in the excitation spectra around 450 nm is the marker that the emitting species are not bound to the protein. The shape of the spectra matches that of free FAD_{ox} in buffer (black lines).

2. Transient absorption (TA) experiments

A 10 kHz, 1 mJ regenerative Ti:Sa amplifier (Legend Elite, Coherent) provides pulses with ~25 fs duration centered around 800 nm. The laser is pumping a tunable optical parametric amplifier (TOPAS, Light Conversion) generating 450 nm pulses with a bandwidth of 15 nm. A pair of chirped mirrors (DCM 12, Laser Quantum) is used for chirp compensation of setup dispersion. A home-built Transient Grating Frequency Resolved Optical Gating (TG-FROG) setup (5) provides a measured pulse duration of ~28 fs.
A fraction of the fundamental Ti:Sa light is focused into a 3 mm thick calcium fluoride (CaF_2) plate (Eksma Optics), which is continuously moved by two motorized translation stages to avoid photo-induced damage of the crystal over time. The emitted white light spans from ~320-750 nm and is collimated with an off-axis parabolic mirror. Remaining fundamental laser light is attenuated with a colored glass filter (FGS580M, Thorlabs).
Pump and probe beams are both simultaneously chopped (MC2000, Thorlabs) with a 2:1 duty cycle chopper wheel on a single shot basis for data acquisition. This generates a sequence of pump-on/probe-on $S_{pu_{on},\,pr_{on}}(\lambda, t_W)$, pump-off/probe-on $S_{pu_{on}pr_{off}}(\lambda)$ and pump-on/probe-off $S_{pu_{off},\,pr_{on}}(\lambda)$ spectra that is then used for evaluating differential transmission spectra. A fast and sensitive low noise line camera (Aviiva EM4, e2v), mounted to a 150 mm grating monochromator (Acton SP-2150, Princeton Instruments, 150 l/mm grating blazed for 500 nm) records 1000 single shot spectra for each pulse delay t_W. From these individual spectra, scattering-corrected differential transmission spectra

$$\frac{\Delta T}{T}(\lambda, t_W) = \frac{S_{pu_{on},\,pr_{on}}(\lambda, t_W) - S_{pu_{on}pr_{off}}(\lambda) - S_{pu_{off},\,pr_{on}}(\lambda)}{S_{pu_{off},\,pr_{on}}(\lambda)} \quad \text{(S1)}$$

are calculated and then averaged. The subtraction of the $S_{pu_{on}pr_{off}}(\lambda)$ spectrum ensures that weak and spurious light scattering contributions on the detector that are induced by the pump pulse are efficiently suppressed. The waiting time delays t_W between pump and probe are changed with a linear translation stage (M531.5IM, Physik Instrumente). A series of delay scans are performed and averaged afterwards.
A temperature-controlled sample chamber, set to 1°C, is continuously moved to minimize effects of photoreduction. The pump and probe beam are focused into the sample under a small angle of ~4° by use of an off-axis parabolic mirror.
TA measurements are performed with 20 nJ pump and ~1 nJ probe pulses at 10 kHz laser repetition rate. The pump pulses are focused to a spot size of ~50x50 µm², giving an excitation fluence of 1 mJ/cm² The polarization of the probe is parallel to the optical table, while the pump has a polarization of 45°. A polarizer mounted on a motorized rotation stage can turn the pump polarization, allowing for parallel and crossed polarization between pump and probe without affecting the pump power.
Experimental data is acquired in crossed and parallel polarization for each scan, from which isotropic differential transmission spectra at magic angle (MA) are calculated as

$$\frac{\Delta T}{T}_{MA} = \frac{1}{3}\left(\frac{\Delta T}{T}_{parallel} + 2\frac{\Delta T}{T}_{crossed}\right) \quad \text{(S2)}$$

These MA spectra are displayed in the main manuscript and used for further analysis.

3. Linearity and sample stability

To ensure that TA experiments are performed in a linear pump-power regime, the dependence of $\Delta T/T$ signal strength on pump pulse energy was recorded for a wild type sample. The spot sizes of pump and probe at their intersection were set to ~50x50 µm².

Fig. S2a shows that the shape of the $\Delta T/T$ spectra at a fixed delay of 2 ps, normalized to their maximum at 450 nm, does not change with pump energy in the range between 5 and 40 nJ. Also the dynamics at selected delays do not change when varying the pump energy in this range. As seen in Fig. 2c, no deviation from a linear pump-power dependence can be observed. This ensures that all reported TA measurements in this manuscript, recorded at a pump energy of 20 nJ, are well within the linear pump-power regime, not exceeding the $\chi^{(3)}$ regime. To test for possible sample degradation during the nonlinear measurements, we have investigated the time evolution of the differential transmission spectrum of a W_BF mutant at a fixed delay of 2 ps. No visible change in $\Delta T/T$ is seen during the course of the 6.5 h measurement. The experimental conditions were the same as for the data shown in Fig. 2 and 4 of the main manuscript. In Fig. S3b, the dynamics of the $\Delta T/T$ signal are displayed at selected probe wavelengths of 450 nm and 550 nm for all 30 scans shown in Fig. S3a. Also here, we find no change of signal over the span of the measurement time, indicating negligible amounts sample degradation during the 6.5 h measurement period, longer than any measurement reported in the main manuscript.

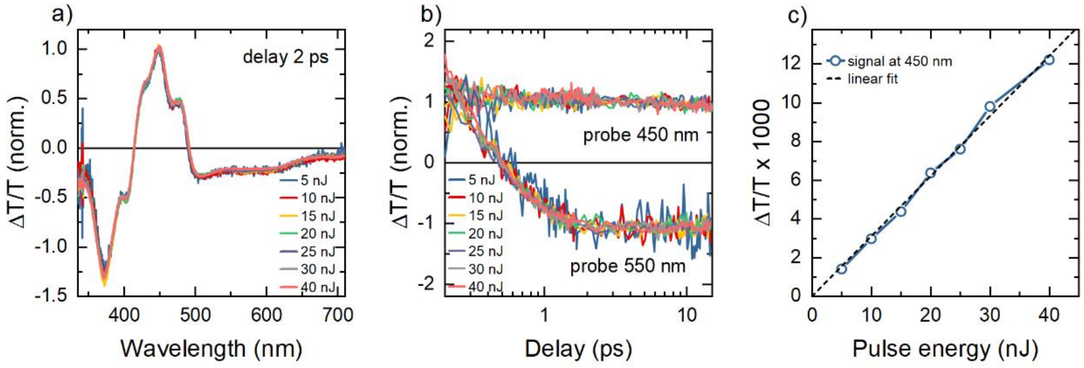

Figure S2: Effect of the pump pulse energy on $\Delta T/T$ signals for wild type *Er*Cry4 protein. a) Normalized differential spectra at a delay of 2 ps and b) normalized dynamics at 450 nm and 550 nm recorded for pump pulse energy ranging from 5 nJ to 40 nJ. c) Differential transmission signal averaged over the delays from 5 ps to 15 ps at 450 nm as a function of the pump pulse energy (blue circles) together with a linear fit through the origin. This linear relationship of signal strength and pump pulse energy shows that the experiments are performed well within the linear pump power regime.

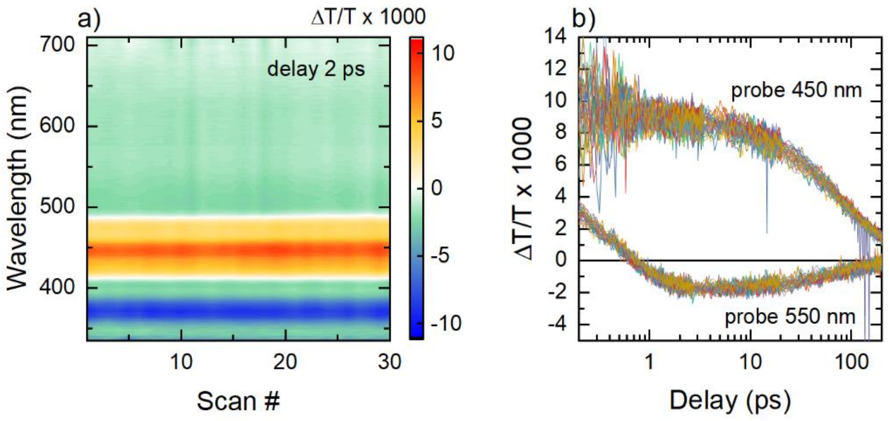

Figure S3: Stability of the differential transmission spectra over 30 delay scans, for a measurement of the W_BF mutant, lasting ~6.5 h. The measurement is performed for parallel pump/probe polarization using 20 nJ pump pulses at 450 nm. a) Raw differential transmission spectra at a delay of 2 ps for all 30 scans show no visible change during the entire measurement. b) The dynamics of all scans at 450 nm and 550 nm further indicate no significant sample degradation during the measurement. Fluctuations at 450 nm within the first ps arise from interferences between the probe and scattered pump pulse, remaining after scattering correction.

4. PFC oxidizer

In all experiments that are shown in the main manuscript, potassium ferricyanide (PFC) has been added as an oxidizing agent to counteract photoreduction of the sample during the measurement (6). Figure S4a shows the linear absorption spectrum of 1 mM PFC in Tris buffer (20% glycerol, 200 mM NaCl, pH 8.0). The main absorption band around ~420 nm has some overlap with the 450 nm pump pulse spectrum.

The addition of ~1.5 mM PFC during sample preparation will oxidize a partially reduced sample. This oxidation may partially or even fully deplete the PFC concentration. In the latter case, additional PFC has been added until the sample shows no residual absorption of reduced FAD. A reduced sample shows a broad FADH absorption band in the range of 500-700 nm, in contrast to a fully reduced sample only containing FAD_{ox}. The amount of PFC remaining in the now fully oxidized sample is then determined using linear absorption measurements. The remaining amount of PFC, which counteracts photo-reduction during the experiments is found to range from ~1.7-2.6 mM in the investigated samples at the start of the measurement.

A TA measurement of 1 mM PFC in buffer under the same experimental conditions as the *Er*Cry4 measurements is shown in Figure S4b. Here, only a very weak differential transmission signal on the order of 0.01% can be observed, decaying within a few ps. This makes possible PFC contributions to the nonlinear signal of the protein measurements negligible.

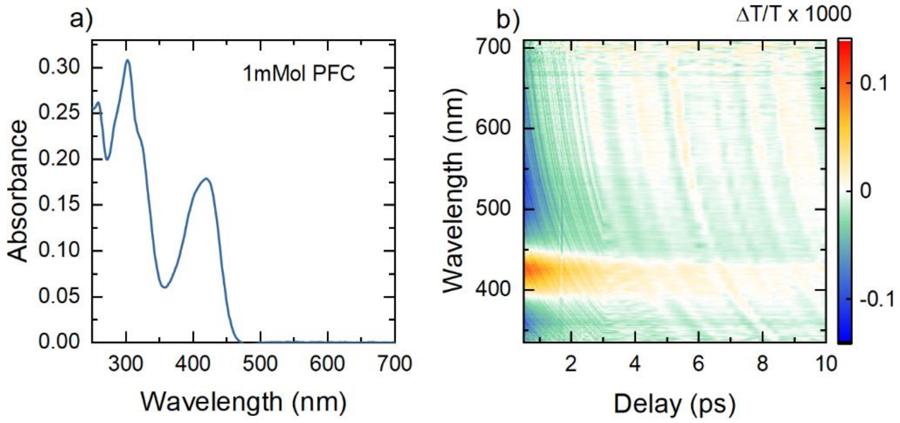

Figure S4: Linear absorption and nonlinear differential transmission spectra of PFC oxidizer. a) Linear absorption spectrum of 1 mM PFC in Tris buffer. b) Probe-chirp-corrected $\Delta T/T$ map of 1 mM PFC in buffer recorded under the same experimental conditions as for the *Er*Cry4 measurements. Note that the differential transmission signal is < 10^{-4}.

5. FAD in buffer

A TA measurement of a 200 µM solution of molecular FAD dissolved in a buffer containing 1 mM PFC is shown in Fig. S5. The sample was measured under the same experimental conditions as used for the *Er*Cry4 measurements. A global analysis of the data results in four DADS spectra that are displayed in Fig. S5d. The spectral shapes of these DADS spectra and their associated decay times closely follow those reported by Brazard et al. (7). Here, the long-lived 3.2 ns component (3.0 ns in Ref. (7)) can be assigned to the relaxation of the open conformer of optically excited FAD, in which the distance between the electron-donating adenine moiety and the electron-accepting isoalloxazine group is so large that an intramolecular electron transfer is efficiently suppressed. The DADS spectrum with a much shorter decay time of 6.6 ps (5.4 ps in Ref. (7)) is assigned to monitor the intramolecular electron transfer between adenine and isoalloxazine in the closed conformer of FAD. Additional DADS spectra with decay times of 1.7 ps and 35 ps (1 ps and 31 ps in Ref. (7), respectively) are attributed to solvation dynamics and to the decay of the FAD dimer, respectively. No effect of PFC on dynamics and spectral shapes can be observed.

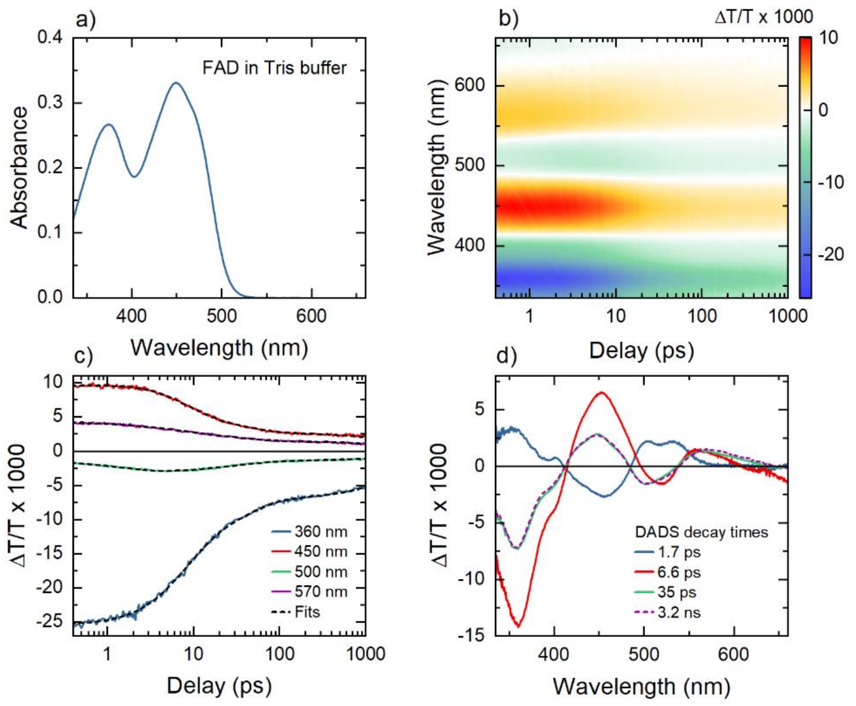

Figure S5: Linear absorption and nonlinear differential transmission of molecular FAD in buffer solution. a) Linear absorption spectrum of 0.2 mM FAD in Tris buffer (20% glycerol, 200 mM NaCl, pH 8.0). b) Probe-chirp-corrected $\Delta T/T$ map of 200 µM FAD in Tris buffer containing 2 mM PFC, measured under the same experimental conditions as the *Er*Cry4 measurements. c) Dynamics of the differential transmission signal for selected wavelengths. The result of the global analysis of the data is superimposed as dashed black lines. d) The four DADS spectra that are obtained from a global analysis of the data and their associated decay times. Spectra and decay times are very similar to those reported in Ref. (7) for a sample with a 0.23 mM concentration. No effects of the PFC oxidizer on the dynamics and on the spectra can be observed.

6. Data preparation and analysis

For each ErCry4 measurement, a measurement of the differential transmission signal of a plain buffer solution was performed. This measurement shows, within +/- 100 fs around time zero and at all probe wavelengths, a well-known and strong coherent scattering contribution that arises from the cross-phase modulation between the chirped few-ps probe pulse and the short pump pulse (8). This coherent scattering signal can be employed to measure the wavelength-dependent zero point of the delay, $t_W = 0$. For this, we have used the method described in (9) to fit the solvent signal. Based on this fit, the origin of the time delay axis was corrected for each TA measurement. Afterwards, the coherent solvent contribution, as obtained from the reference measurement, has been subtracted from the protein measurement. To avoid any spurious, residual solvent contribution in the measurements discussed in this manuscript, we have discarded the TA data for delays below 200 fs from the data analysis.

To investigate the ErCry4 wild type and mutant datasets, a global analysis was performed by use of a Matlab-based toolbox (10). To obtain decay associated difference spectra (DADS), only monoexponential decays were used as mathematical model functions. Each DADS component, $\mathrm{DADS_i}(\lambda), i = 1, \dots, n$, represents the spectral amplitude of the signal contribution that is decaying with a time constant τ_i. In this way, the global fit will reproduce the complete, experimentally measured data set by a sum over all $\mathrm{DADS_i}(\lambda)$ spectra multiplied by an exponential decay with decay time τ_i

$$\frac{\Delta T}{T}(\lambda, t_W) = \sum_{i=1}^{n} \mathrm{DADS_i}(\lambda)\, e^{-\frac{t_W}{\tau_i}} \tag{S3}$$

The i-th term of this sum, $\mathrm{DADS_i}(\lambda)\mathrm{e}^{-\mathrm{tw}/\tau_i}$, is the time-dependent differential transmission spectrum associated with the relaxation component i. This representation in terms of independent components with monotonically increasing decay times accurately describes the dynamics expected from a sequential rate equation model.

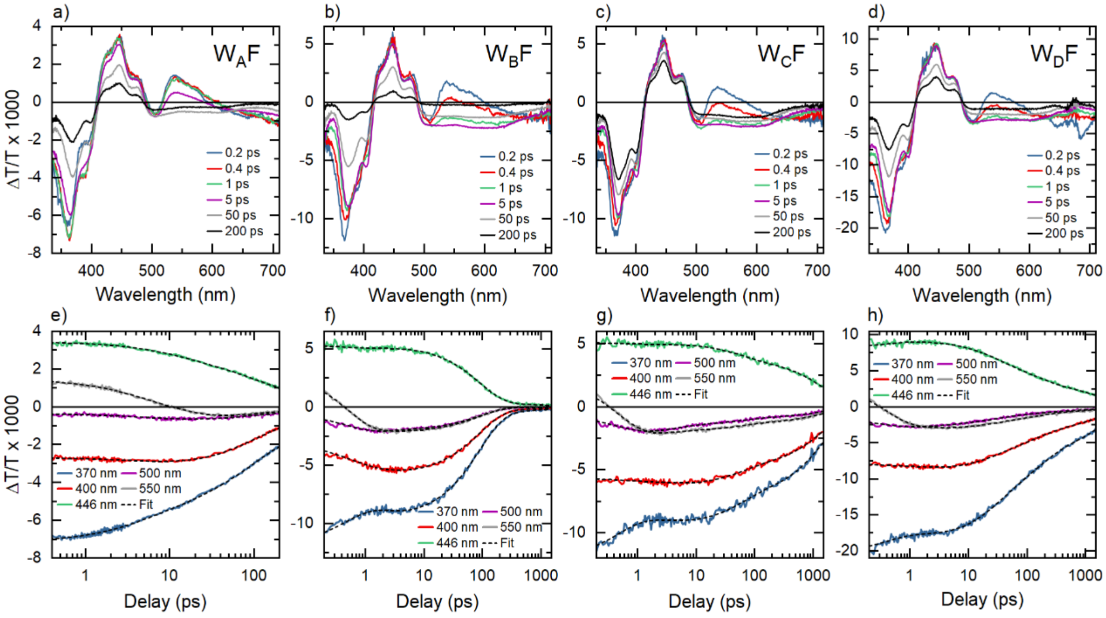

Figure S6: a)-d): Differential transmission spectra of all four investigated *Er*Cry4 mutants at selected delays. The spectra are cross-sections through the data presented in Fig. 2 of the main manuscript. e)-h): Dynamics at selected wavelengths for the four mutants (solid lines). The fits obtained from the global analysis (black dashed lines) show excellent agreement with the experimental data.

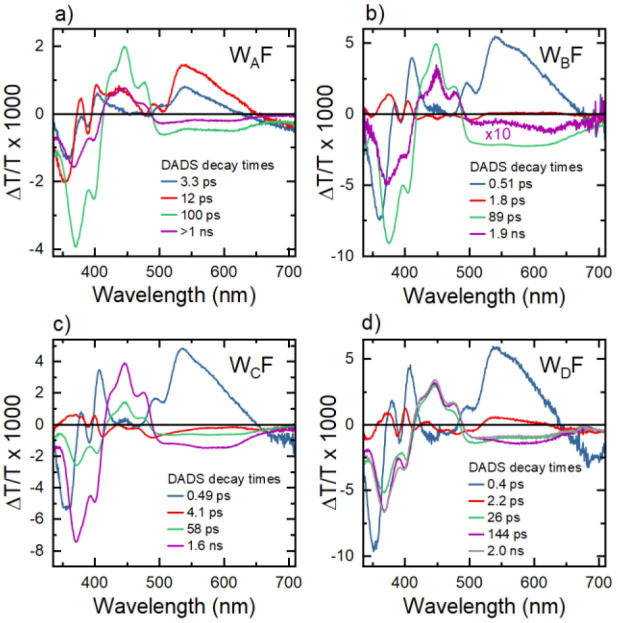

Figure S7: DADS spectra resulting from a global analysis of the differential transmission maps recorded for the four *Er*Cry4 mutants (Fig. 2 of the main manuscript). The global analysis results in a minimal number of physically meaningful decay components with different differential transmission spectra and associated decay times that are depicted in the individual subpanels. These DADS spectra are then used to calculate the EADS spectra of the four mutants that are shown in Figs. 2e-h of the main manuscript.

The DADS spectra that result from the global analysis for all mutants and the wild type are shown in Fig. S7. The datasets were modeled using the smallest number of decays necessary to fully describe the dynamics of the data at all wavelengths. The correctness of this number was judged by the appearance of temporally and spectrally flat and unstructured residuals. For wild type ErCry4 and the W_DF mutant five and for the W_AF, W_BF and W_CF mutants four exponential decays were needed to represent the measured data. Figure S6e-h shows the fits resulting from the global analysis compared to the experimental data dynamics at selected wavelengths. A convincing agreement between fit and data validates the DADS obtained by the global analysis.

These DADS spectra may either reflect the exponential decay or the rise of a certain spectral component in the data. As such, their physical interpretation may sometimes be challenging when spectral components that rise in time overlap with decaying components. This is indeed the case for the present samples where some spectral signatures of the rise in radical pair concentration overlap with decaying signals from oxidized flavin. The physical interpretation of the spectra may be facilitated by transforming them into evolution-associated difference spectra (EADS) that are defined (11) as

$$\mathrm{EADS_k}(\lambda) = \sum_{i=k}^{n} \mathrm{DADS_i}(\lambda). \tag{S4}$$

The first spectrum, $\mathrm{EADS_1}$, represents the sum over all DADS spectra and, thus, the differential transmission spectrum at delay zero, $\Delta T/T(\lambda, t_W = 0)$. The k-th spectrum, $\mathrm{EADS_k}, k > 1$, then gives a differential transmission spectrum which is the difference between $\Delta T/T(t_W = 0)$ and the sum of the first $k - 1$ DADS spectra. Provided that the decay constants are sufficiently different, this approximately represents the shape of the differential transmission at a delay shortly after these $k - 1$ relaxation steps have been completed.

The EADS spectra are then used to extract spectra for different electronic states of FAD (FAD_{ox}, FAD^*_{ox} and $FAD^{\bullet-}$), as well as for the different tryptophans (Trp_A, Trp_B, Trp_C). The absorption spectra of the excited flavin, $A_{\mathrm{FAD^*_{ox}}}$, and the third radical pair A_{RP3} were obtained by removing the measured absorbance $A_{\mathrm{FAD_{ox}}}$ from $\mathrm{EADS_1}$ and $\mathrm{EADS_5}$, which were converted from differential transmission $\Delta T/T$ to differential absorbance ΔA using

$$A_{\mathrm{FAD^*_{ox}}}(\lambda) = \log_{10}\left(1 - \mathrm{EADS_1}(\lambda)\right) + nA_{\mathrm{FAD_{ox}}}, \text{ and} \tag{S5}$$

$$A_{\mathrm{RP3}}(\lambda) = A_{\mathrm{FAD^{\bullet-}}}(\lambda) + A_{\mathrm{Trp_CH^{\bullet+}}}(\lambda) = \log_{10}\left(1 - \mathrm{EADS_5}(\lambda)\right) + nA_{\mathrm{FAD_{ox}}}. \tag{S6}$$

The scaling factor n is is chosen as 0.025 such that the obtained spectra are flat and unstructured at the peaks around 450 nm, similar to procedures described in (12, 13).
Literature spectra of $TrpH^+$ show mainly absorption above ~500 nm and below ~380 nm (14), while $FAD^{\bullet-}$ only absorbs at wavelengths below ~500 nm (15, 16). Thus, to disentangle the absorbance spectrum A_{RP3} into its components $A_{\mathrm{FAD^{\bullet-}}}$ and $A_{\mathrm{Trp_CH^{\bullet+}}}$, a sum of 11 Gaussian spectra is fitted to A_{RP3}, where 4 Gaussians represent the plateau for wavelengths above ~500 nm, taken as the $Trp_CH^{\bullet+}$ absorbance. After subtracting $A_{\mathrm{Trp_CH^{\bullet+}}}$ from A_{RP3}, the remaining $FAD^{\bullet-}$ absorbance is used to extract the $Trp_BH^{\bullet+}$ and $Trp_AH^{\bullet+}$ spectra from $\mathrm{EADS_4}$ and $\mathrm{EADS_2}$, respectively.
Absorbance is converted to molar extinction ε using the experimentally measured FAD_{ox} absorbance and a reference value of 11300 $M^{-1}cm^{-1}$ for the molar extinction of FAD_{ox} at a wavelength of 450 nm (7, 11, 12). This approach results in the species spectra depicted in Fig. 3c, which are very similar to those reported by Kutta et al. (12).

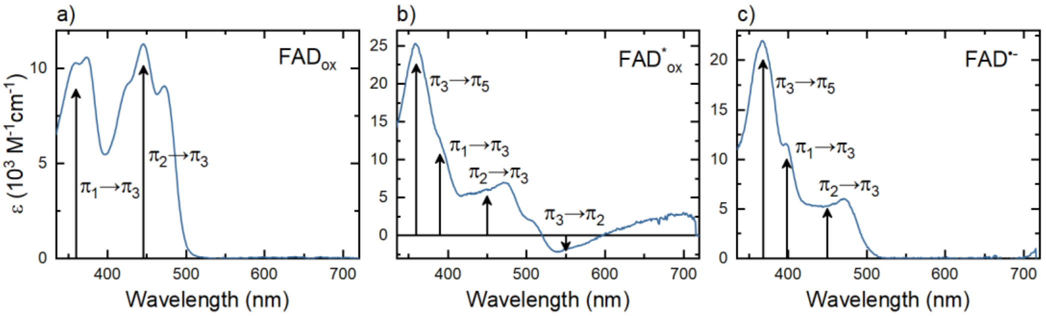

Figure S8: Spectral assignment of the differential transmission spectra of a) FAD$_{ox}$, b) FAD$^*_{ox}$ and c) FAD$^{•-}$ as obtained from the W$_D$F dataset. The assignment is based on quantum-chemical calculations recently reported by Schwinn et al. (15). The corresponding intramolecular transitions for FAD$_{ox}$ and FAD$^{•-}$ are marked with black arrows. The molecular orbitals that are involved in the FAD$^*_{ox}$ absorption are assumed to be the same as those for FAD$^{•-}$. An additional stimulated emission peak ($\pi_3 \rightarrow \pi_2$) appears in FAD$^*_{ox}$ at around 550 nm.

7. Additional simulation data

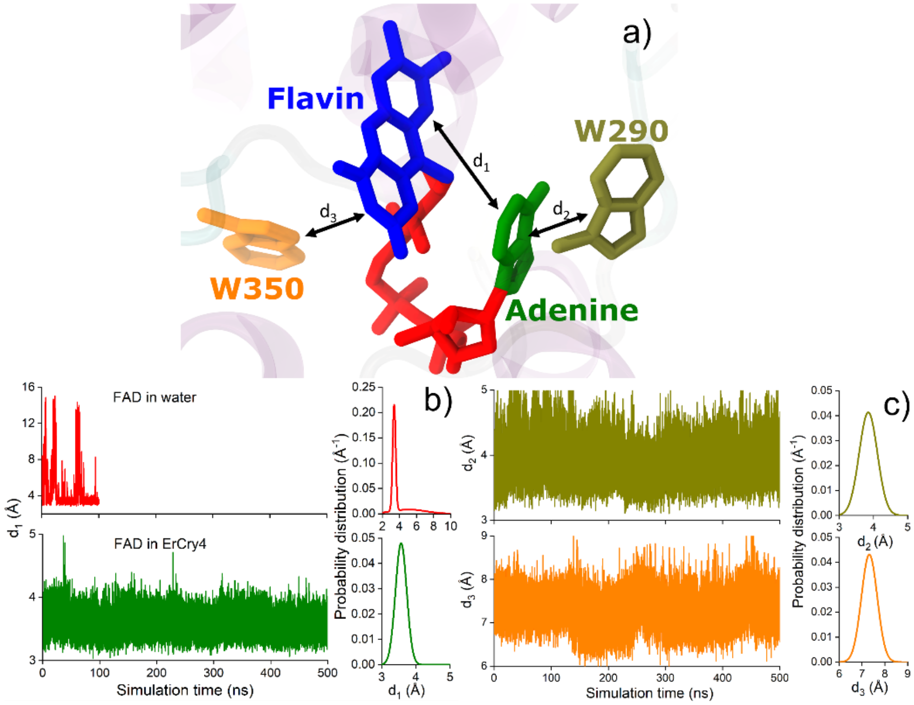

Figure S9: (a) Flavin adenine dinucleotide (FAD) cofactor with the indicated internal edge-to-edge distances between the flavin and adenine (d$_1$) cofactors, the edge-to-edge distance between adenine and Trp290 residue (d$_2$) and the edge-to-edge distance between the flavin and the Trp350 residue (d$_3$). (b) Flavin-adenine edge-to-edge distance for solvated FAD in water and embedded in *Er*Cry4 respectively with the corresponding probability density distributions. (c) Edge-to-edge distances between adenine and the Trp290 residue (d2) and flavin and the Trp350 residue (d$_3$) in *Er*Cry4, see Fig. 5. Time evolution of the two distances as well as the probability density distribution of the distances are shown.

8. SI references

1. D. C. Lünemann *et al.*, Distinguishing between coherent and incoherent signals in excitation-emission spectroscopy. *Opt Express* **29**, 24326-24337 (2021).

2. D. Brida, C. Manzoni, G. Cerullo, Phase-locked pulses for two-dimensional spectroscopy by a birefringent delay line. *Opt. Lett.* **37**, 3027-3029 (2012).

3. A. Perri *et al.*, Hyperspectral imaging with a TWINS birefringent interferometer. *Opt Express* **27**, 15956-15967 (2019).

4. C. M. Andersen, R. Bro, Practical aspects of PARAFAC modeling of fluorescence excitation-emission data. *J Chemometr* **17**, 200-215 (2003).

5. J. N. Sweetser, D. N. Fittinghoff, R. Trebino, Transient-grating frequency-resolved optical gating. *Opt Lett* **22**, 519-521 (1997).

6. J. J. Xu *et al.*, Magnetic sensitivity of cryptochrome 4 from a migratory songbird. *Nature* **594**, 535-540 (2021).

7. J. Brazard *et al.*, New Insights into the Ultrafast Photophysics of Oxidized and Reduced FAD ins Solution. *J Phys Chem A* **115**, 3251-3262 (2011).

8. K. Ekvall *et al.*, Cross phase modulation artifact in liquid phase transient absorption spectroscopy. *J Appl Phys* **87**, 2340-2352 (2000).

9. B. Baudisch (2018) Time resolved broadband spectroscopy from UV to NIR.

10. M. Rabe, Spectram: A MATLAB® and GNU Octave Toolbox for Transition Model Guided Deconvolution of Dynamic Spectroscopic Data. *Journal of Open Research Software* **8**, 13 (2020).

11. F. Lacombat *et al.*, Ultrafast Oxidation of a Tyrosine by Proton-Coupled Electron Transfer Promotes Light Activation of an Animal-like Cryptochrome. *J Am Chem Soc* **141**, 13394-13409 (2019).

12. R. J. Kutta, N. Archipowa, N. S. Scrutton, The sacrificial inactivation of the blue-light photosensor cryptochrome from Drosophila melanogaster. *Phys Chem Chem Phys* **20**, 28767-28776 (2018).

13. R. J. Kutta, N. Archipowa, L. O. Johannissen, A. R. Jones, N. S. Scrutton, Vertebrate Cryptochromes are Vestigial Flavoproteins. *Sci Rep-Uk* **7**, 44906 (2017).

14. S. Solar, N. Getoff, P. S. Surdhar, D. A. Armstrong, A. Singh, Oxidation of Tryptophan and N-Methylindole by N3., Br2-, and (Scn)2- Radicals in Light-Water and Heavy-Water Solutions - a Pulse-Radiolysis Study. *J Phys Chem-Us* **95**, 3639-3643 (1991).

15. K. Schwinn, N. Ferre, M. Huix-Rotllant, UV-visible absorption spectrum of FAD and its reduced forms embedded in a cryptochrome protein. *Phys Chem Chem Phys* **22**, 12447-12455 (2020).

16. Y. T. Kao *et al.*, Ultrafast dynamics of flavins in five redox states. *J Am Chem Soc* **130**, 13132-13139 (2008).
